# Supplementary material for: GPU-accelerated homology search with MMseqs2
Source: Nat Methods. 2025 Sep 18;22(10):2024–7. doi: 10.1038/s41592-025-02819-8 (PMC12510879; doi:10.1038/s41592-025-02819-8)
Supplement: Supplementary file 4 — Binary for introduced software with execution examples and instructions. [file 41592_2025_2819_MOESM4_ESM.zip › mmseqs/userguide.pdf]

MMseqs2 User Guide

Martin Steinegger, Milot Mirdita, Eli Levy Karin, Lars von den Driesch, Clovis Galiez, Johannes Söding

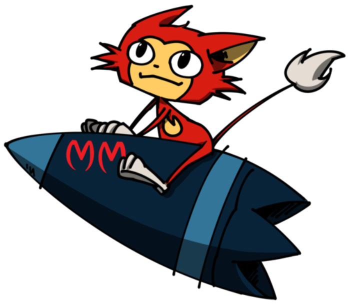

Contents

Summary . . . 5
System requirements . . . 6
Installation . . . 7
- Install MMseqs2 for Linux . . . 7
- Install MMseqs2 for macOS . . . 9
- Install MMseqs2 for Windows (preview) . . . 11
- Use the Docker container . . . 12
- Set up the Bash/Zsh command completion . . . 12
- Customizing compilation through CMake . . . 13

Getting started . . . 14
- Usage of MMseqs2 modules . . . 14
- Easy Workflows in MMseqs2 . . . 15
- Using MMseqs2 Workflows and Modules . . . 15
- Downloading databases . . . 16
- Searching . . . 17
- Clustering . . . 20
- Linclust . . . 20
- Updating a clustered database . . . 21

Overview of folders in MMseqs2 . . . 21
Overview of important MMseqs2 Modules . . . 21
Description of workflows . . . 23
- Batch sequence searching using `mmseqs search` . . . 23
- Expanded cluster searches . . . 24
- Translated sequence searching . . . 25
- Mapping very similar sequences using `mmseqs map` . . . 26
- Clustering databases using `mmseqs cluster` or `mmseqs linclust` . . . 27
- Linear time clustering using `mmseqs linclust` . . . 33
- Updating a clustering database using `mmseqs clusterupdate` . . . 35
- Taxonomy assignment . . . 36
- Reciprocal best hit using `mmseqs rbh` . . . 48

Description of core modules 48
- Computation of prefiltering scores using `mmseqs prefilter` 48
- Local alignment of prefiltered sequence pairs using `mmseqs align` 51
- Clustering sequence database using `mmseqs clust` 52

File formats 52
- MMseqs2 database format 52
- Manipulating databases 54
- Sequence database format 54
- Prefiltering format 55
- Alignment format 56
- Clustering format 59
- Taxonomy format 60
- Profile format 61
- Identifier parsing 64

Optimizing sensitivity and consumption of resources 64
- Prefiltering module 64
- Alignment module 67
- Clustering module 68
- Workflows 69

How to run MMseqs2 on multiple servers using MPI 69

How to run MMseqs2 on multiple servers using batch systems 71

Frequently Asked Questions 71
- How to set the right alignment coverage to cluster 71
- How do parameters of CD-HIT relate to MMseqs2 73
- How does MMseqs2 compute the sequence identity 74
- How to restart a search or clustering workflow 75
- How to control the speed of the search 76
- How to find the best hit the fastest way 76
- How does MMseqs2 handle low complexity 77
- How to redundancy filter sequences with identical length and 100% length overlap 77
- How to add sequence identities and other alignment information to a clustering result 77
- How to run external tools for each database entry 78
- How to compute a multiple alignment for each cluster 78
- How to manually cascade cluster 78
- How to cluster using profiles 79
- How to create a HHblits database 79

How to create a target profile database (from PFAM) 80
How to cluster a graph given as tsv or m8 file 81
How to search small query sets fast 82
What is the difference between the map and search workflow 83
How to build your own MMseqs2 compatible substitution matrices 83
How to create a fake prefiltering for all-vs-all alignments 83
How to compute the lowest common ancestor (LCA) of a given set of sequences 84
Workflow control parameters 85
Search workflow 85
Clustering workflow 86
Updating workflow 86
Environment variables used by MMseqs2 87
External libraries used in MMseqs2 87
License terms 88

Summary

MMseqs2 (Many-against-Many searching) is a software suite to search and cluster huge sequence sets. MMseqs2 is open source GPL-licensed software implemented in C++ for Linux, Mac OS and Windows. MMseqs2 is designed to run on multiple cores and servers, making it highly scalable. MMseqs2 matches the sensitivity of BLAST, but with several orders of magnitude faster speed. It can perform profile searches, like PSI-BLAST, but with a speed advantage of up to 400 times faster.

While MMseqs2 includes many more modules, the prefiltering and alignment modules are the core modules that underpin its algorithm. The prefiltering module performs a rapid and sensitive double consecutive k-mer matching-based calculation of similarities between sequences in a query database and a target database, followed by an ungapped alignment. The alignment module then performs a vectorized Smith-Waterman alignment of sequences that pass a cut-off score in the prefiltering module. Both modules are parallelized to use all available computer cores, resulting in a highly efficient software suite. With its combination of sensitivity and speed, MMseqs2 can effectively search predicted ORFs in vast metagenomics datasets against the entire UniProtKB or NCBI-NR databases and assign functional clusters and taxonomic clades to reads that other software may be unable to map.

MMseqs2's clustering module is highly efficient at grouping similar sequences into clusters. It takes the similarity graph generated from the comparison of a sequence set with itself in the prefiltering or alignment modules as its input. Furthermore, MMseqs2 provides an updating workflow that allows for the addition of new sequences to an existing clustering while maintaining stable cluster identifiers, eliminating the need to recluster the entire sequence set. We use MMseqs2 to regularly update

versions of the UniProtKB database, which have been clustered down to a 30% sequence similarity threshold. This database is accessible at uniclust.mmseqs.com.

System requirements

MMseqs2 is compatible with modern UNIX-based operating systems and has been thoroughly tested on Linux and macOS. While we do provide a version of MMseqs2 for Windows, we recommend that users utilize the Linux version through Windows Subsystem for Linux (WSL) for the best performance and stability.

MMseqs2 utilizes OpenMP to enable multi-core processing and takes advantage of the SIMD capabilities of the host system. For optimal performance, we recommend a system with the AVX2 instruction set, although MMseqs2 can also run on systems with SSE4.1 or even SSE2. Additionally, MMseqs2 is compatible with the PPC64LE and ARM64 processor architectures, which require support for the AltiVec and NEON SIMD instruction sets, respectively.

When searching large databases, MMseqs2 may require a significant amount of main memory (see section memory requirements). To address this issue, we offer an option for limiting memory usage at the expense of longer runtimes. The database is split into chunks, with the program only retaining one chunk in memory at a time (see Database splitting runtime slowdown). If clustering large databases containing tens of millions of sequences, ensure that you have sufficient free disk space (~500 GB). In the section Optimizing sensitivity and consumption of resources, we will explore the runtime, memory, and disk space requirements of MMseqs2 and discuss ways to optimize resource usage for large databases.

To determine if MMseqs2 is supported on your system, run the following commands based on your operating system:

Check system requirements under Linux

```
[ $(uname -m) = "x86_64" ] && echo "64bit: Yes" || echo "64bit: No"
grep -q avx2 /proc/cpuinfo && echo "AVX2: Yes" || echo "AVX2: No"
grep -q sse4_1 /proc/cpuinfo && echo "SSE4.1: Yes" || echo "SSE4.1: No"
# for very old systems which support neither SSE4.1 or AVX2
grep -q sse2 /proc/cpuinfo && echo "SSE2: Yes" || echo "SSE2: No"
```

Note: MMseqs2 also supports Linux systems running on ARM64 (aarch64), so disregard the commands above for these systems.

Check system requirements under macOS

```
[ $(uname -m) = "x86_64" ] && echo "64bit: Yes" || echo "64bit: No"
sysctl machdep.cpu.leaf7_features | grep -q AVX2 && echo "AVX2: Yes" || echo "AVX2:
↪  No"
sysctl machdep.cpu.features | grep -q SSE4.1 && echo "SSE4.1: Yes" || echo "SSE4.1:
↪  No"
```

Note: MMseqs2 supports all macOS ARM (M1/M2) CPUs, so disregard the commands above for these systems.

Check system requirements under Windows

If MMseqs2 is executed on an unsupported Windows system, the `mmseqs.bat` script will print an error message. On a supported system, the script will execute the appropriate MMseqs2 version and forward all parameters.

Installation

MMseqs2 can be installed on Linux, macOS, or Windows by:

(1) Downloading a statically compiled version (see below).

(2) Compiling from source (see below).

(3) Using Homebrew (on macOS or Linux:

```
brew install mmseqs2
```

(4) Using bioconda:

```
conda install -c conda-forge -c bioconda mmseqs2
```

(5) Using Docker.

```
# without GPU support (arm64 and x86_64)
docker pull ghcr.io/soedinglab/mmseqs2

# with GPU support (x86_64 only)
docker pull ghcr.io/soedinglab/mmseqs2:master-cuda12
```

Alternatively use the MMseqs2 Biocontainer.

Install MMseqs2 for Linux

Install with Homebrew

You can use also use Homebrew on Linux:

```
brew install mmseqs2
```

Install static Linux version

To install the latest MMseqs2 version on Linux, follow these steps:

Download the appropriate static binary package for your system based on the available instruction sets. For systems supporting AVX2, use the following command:

```
wget https://mmseqs.com/latest/mmseqs-linux-avx2.tar.gz
```

For systems supporting SSE4.1, use this command:

```
wget https://mmseqs.com/latest/mmseqs-linux-sse41.tar.gz
```

For very old systems with only support for SSE2, use this command:

```
wget https://mmseqs.com/latest/mmseqs-linux-sse2.tar.gz
```

For GPU-accelerated searches, you can download the following binary:

```
wget https://mmseqs.com/latest/mmseqs-linux-gpu.tar.gz
```

Except GPU-support, this is the same as the AVX2 build above. However, this is not a fully static binary. It can only be used on systems with glibc >= 2.29. This does not require the CUDA toolkit to be installed, only nvidia driver >=525.60.13 is required.

Extract the downloaded archive:

```
tar xvzf mmseqs-linux-*.tar.gz
```

Add the MMseqs2 binary directory to your `PATH` environment variable by running the following command:

```
export PATH=$(pwd)/mmseqs/bin/:$PATH
```

Static binaries for ARM64 and PPC64LE can also be found at https://mmseqs.com/latest.

Compile from source under Linux

Compiling MMseqs2 from source can improve its performance by optimizing it for your specific system. To compile MMseqs2 from source, follow these steps:

```
git clone https://github.com/soedinglab/MMseqs2.git
cd MMseqs2
mkdir build
cd build
```

```
cmake -DCMAKE_BUILD_TYPE=RELEASE -DCMAKE_INSTALL_PREFIX=. ..
make -j8
make install
export PATH=$(pwd)/bin/:$PATH
```

Please note that you will need git, g++ (version 4.9 or higher), and cmake (version 2.8.12 or higher) installed on your system to compile MMseqs2 from source. See the Customizing compilation through CMake section if you want to customize the compilation process, including cross-compiling.

Compile from source for Linux with GPU support

MMseqs2 has gained for support for GPU-accelerated protein sequence and profile search with release 16. This requires an NVIDIA GPU of the Ampere generation or newer for full speed, however, also works at reduced speed for Turing-generation GPUs. We have not tested MMseqs2-GPU on older GPU microarchitectures (e.g. Pascal or Volta) and do not currently support them. We recommend to install CUDA (>= 12.4) dependencies through Conda. The command below compiles code for multiple GPU generations. To speed up compilation, you can use `-DCMAKE_CUDA_ARCHITECTURES="native"`, to compile the code for the installed GPUs only.

```
conda create -n nvcc -c conda-forge cuda-nvcc cuda-cudart-dev cuda-version=12.6 cmake
conda activate nvcc
mkdir build && cd build
cmake -DCMAKE_BUILD_TYPE=RELEASE -DCMAKE_INSTALL_PREFIX=. -DENABLE_CUDA=1
↪  -DCMAKE_CUDA_ARCHITECTURES="75;80;86;89;90" ..
make -j8
make install
export PATH=$(pwd)/bin/:$PATH
```

See the GPU-accelerated search section on how to use MMseqs2-GPU.

Install MMseqs2 for macOS

Install with Homebrew

To install the latest stable version of MMseqs2 on macOS, you can use Homebrew by executing the following command:

```
brew install mmseqs2
```

This command will also install the bash completion for MMseqs2, although you may need to execute `brew install bash-completion` first.

Install static macOS version

To install a static version of MMseqs2 on macOS, you can download the newest commit using our statically compiled binaries. If you do not have wget installed, you can install it using Homebrew with brew install wget. The static binary includes versions for both SSE4.1, AVX2, and ARM (for M1/M2 Macs), and macOS will automatically select the appropriate version.

To install MMseqs2 on macOS, follow these steps:

```
wget https://mmseqs.com/latest/mmseqs-osx-universal.tar.gz
tar xvzf mmseqs-osx-universal.tar.gz
export PATH=$(pwd)/mmseqs/bin/:$PATH
```

This will download and extract the latest version of MMseqs2 and set the `PATH` variable so that the `mmseqs` command is available from the command line.

Compile from source under macOS

Compiling under Clang

To compile MMseqs2 with (Apple-)Clang, you need to install either Xcode or the Command Line Tools. You also need `libomp`. We recommend installing it using Homebrew:

```
brew install cmake libomp zlib bzip2
```

Currently, CMake does not correctly identify paths to `libomp`. To compile MMseqs2, use the script in `util/build_osx.sh`. The resulting binary will be placed in `OUTPUT_DIR/mmseqs`.

To compile MMseqs2 with Clang, follow these steps:

```
./util/build_osx.sh PATH_TO_MMSEQS2_REPO OUTPUT_DIR
```

Replace `PATH_TO_MMSEQS2_REPO` with the path to the MMseqs2 repository on your computer, and replace `OUTPUT_DIR` with the desired output directory.

Compiling using GCC

To compile MMseqs2 using GCC, please install the following packages with Homebrew:

```
brew install cmake gcc@12 zlib bzip2
```

After installing the necessary packages, use the following `cmake` command:

```
CC="gcc-12" CXX="g++-12" cmake -DCMAKE_BUILD_TYPE=Release -DCMAKE_INSTALL_PREFIX=. ..
make
```

This command sets the `CC` and `CXX` environment variables to use the `gcc-12` and `g++-12` compilers, respectively. It also sets the build type to `Release` and the installation prefix to the current directory.

Install MMseqs2 for Windows (preview)

If you are using Windows 10 or later, we recommend installing the Windows Subsystem for Linux (WSL) to use MMseqs2. To install WSL, please refer to Microsoft's documentation.

After installing WSL2 and rebooting, you can follow the Linux instructions above to install and use MMseqs2 on Windows. Please note that the WSL2-based MMseqs2 should be faster than the Cygwin-based MMseqs2 described below.

Install static Windows version

If you cannot use WSL2, you can download either a stable release from our page or download a statically compiled version from the latest Git commit at:

If you cannot use WSL2, you can download a stable release from our GitHub Releases page or a statically compiled version from the latest Git commit at:

```
https://mmseqs.com/latest/mmseqs-win64.zip
```

Download and unzip it at a convenient location. Inside you will find the `mmseqs.bat` wrapper script, which should be used to substitute all calls to `mmseqs` in the remainder of this document, and a `bin` folder with all dependencies of the MMseqs2 Windows version. Please keep the `mmseqs.bat` script always one folder above the `bin` folder, or it will not be able to correctly identify its dependencies anymore.

The Windows build also contains the SSE4.1, and AVX2 versions. The `mmseqs.bat` script will automatically choose the correct one.

Compile from source under Windows

To compile MMseqs2 on Windows, you will need to use the Cygwin environment and Busybox to provide all the necessary dependencies. First, you need to install the following packages from Cygwin:

```
bash xxd cmake make gcc-g++ zlib-devel libbz2-devel busybox-standalone binutils
```

After installing the packages, you can use a similar workflow as the `util/build_windows.sh` script to build MMseqs2 on Windows.

Use the Docker container

You can pull the official docker container from the our Github Container Registry by running: The MMseqs2 Docker container can be pulled from the Github Container Registry using the following command:

```
docker pull ghcr.io/soedinglab/mmseqs2
```

To use the MMseqs2 Docker container, you must mount a folder from the host system into the container using the `-v` argument. The following command mounts the current working directory under /app inside the container and runs the easy-search command:

```
docker run -v "$(pwd):/app" ghcr.io/soedinglab/mmseqs2 easy-search /app/QUERY.fasta
 ↪  /app/DB.fasta /app/result.m8 /app/tmp
```

Building the Docker container

The Dockerfile used to build the MMseqs2 Docker container requires `docker buildx`, which should be installed with a regular Docker installation (version 19.03 or higher). To build the Docker container from the Git repository, follow these steps:

```
git clone https://github.com/soedinglab/MMseqs2.git
cd MMseqs2
docker buildx build -t mmseqs2 .
```

This will create a new Docker image with the tag mmseqs2 that you can use to run MMseqs2 in a containerized environment.

Set up the Bash/Zsh command completion

MMseqs2 comes with a Bash command and parameter auto completion feature, which makes it easy to navigate and execute MMseqs2 modules, workflows and parameters by providing a list of available parameters or completing partially typed module/workflow names. To enable this feature, add the following lines to your `$HOME/.bashrc`:

```
if [ -f /Path to MMseqs2/util/bash-completion.sh ]; then
    source /Path to MMseqs2/util/bash-completion.sh
fi
```

The Bash completion can also be used with Zsh by loading the `bashcompinit` compatibility shim first. Add the following lines to your `$HOME/.zshrc`:

```
if [ -f /Path to MMseqs2/util/bash-completion.sh ]; then
    autoload -U +X compinit && compinit
    autoload -U +X bashcompinit && bashcompinit
    source /Path to MMseqs2/util/bash-completion.sh
fi
```

Customizing compilation through CMake

Compiling MMseqs2 from source results in binaries that are optimized for the system on which MMseqs2 was compiled. Using these binaries on another system may lead to errors (e.g., `Invalid instruction`) or degraded performance. You can customize the compilation process by specifying the appropriate flags during the cmake call for your system. The following flags are available:

- `-DHAVE_AVX2=1`: for AMD/Intel x64 systems with support for AVX2 instructions.
- `-DHAVE_SSE4_1=1`: for AMD/Intel x64 systems with support for SSE4.1 instructions
- `-DHAVE_SSE2=1`: for old AMD/Intel x64 systems with support for SSE2 instructions.
- `-DHAVE_POWER9=1`: for PPC64LE systems with support for POWER9 AltiVec instructions.
- `-DHAVE_POWER8=1`: for PPC64LE systems with support for POWER8 AltiVec instructions.
- `-DHAVE_ARM8=1`: for ARM64 systems with support for NEON instructions.

You can also disable the automatic setup of architecture-specific compilation flags with `-DNATIVE_ARCH=0`. Use `-DCMAKE_C_FLAGS` and `-DCMAKE_CXX_FLAGS` to set the flags for your system. This is useful in combination with the `-mtune`/`-march`/`-mcpu` flags of GCC/Clang if you know exactly what system your binary will later run on.

To disable the use of the ips4o sorting library, which requires atomic compare exchange instructions for 16-byte/128-bit values, you can use `-DDISABLE_IPS4O=1`. Usually, MMseqs2 automatically enables/disables ips4o depending on the system, and this flag should not be changed.

You can change the MMseqs2 version string, which is printed in help texts and by the version module, with the `-DVERSION_OVERRIDE` parameter. This is useful for packaging MMseqs2 to set a custom version string for the distribution name.

The `-DHAVE_SANITIZER=1`parameter enables additional build types for debugging MMseqs2. Refer to the developer guide for more information.

The `-DENABLE_CUDA=1` parameter builds the GPU version of MMseqs2.

The `-DHAVE_MPI=1` parameter builds the MPI version of MMseqs2. See the How to run MMseqs2 on multiple servers using MPI section for more information.

MMseqs2 requires OpenMP support for compilation, and it will refuse to compile without it. To build a single-threaded version of MMseqs2, use the `-DREQUIRE_OPENMP=0` parameter during the

cmake invocation.

MMseqs2 requires zstd to compile. You can use the system-provided zstd instead of the bundled one by using -DUSE_SYSTEM_ZSTD=1 during the cmake invocation.

Getting started

Here we explain how to run a search for sequences matches in the query database against a target database and how to cluster a sequence database. Test data (a query and a target database for the sequence search and a database for the clustering) are provided in the examples folder.

Usage of MMseqs2 modules

Calls to MMseqs2 modules and workflows follow the structure:

```
mmseqs module input_db output_db args [options]
```

The module parameter specifies one of the many available modules or workflows, such as search, easy-search, createdb, and more. The args parameter specifies other obligatory paths or filenames that the module needs, and options can be provided to modify the behavior of the module or change parameter settings. Workflows and modules are the same from the user perspective; workflows are shell scripts that chain together elementary MMseqs2 modules and other workflows. A complete list of available modules can be obtained by running mmseqs -h.

You can get information about any MMseqs2 module by simply calling it without arguments and options, e.g.

```
mmseqs createdb
```

This will give a short description, the usage text, and a list of the most important options. The usage explains the syntax of the module. For example, mmseqs createdb will give you its usage string as:

```
Usage: <i:fastaFile1[.gz]> ... <i:fastaFileN[.gz]> <o:sequenceDB> [options]
```

<...> denote files, <i:...> and <o:...> denote input and output files, respectively, brackets [] indicate that what is enclosed is optional, whereas everything not enclosed in [] cannot be omitted.

An extended list of parameters can be shown by adding the -h flag to the MMseqs2 call:

```
mmseqs createdb -h
```

MMseqs2 workflows combine and chain these single-purpose modules into larger tasks, such as searching, clustering or taxonomic annotation. When you pass -h to a workflow it will show a

combined list of parameters of all individual modules. Some parameter combinations might be non-sensical or conflicting. Generally, be careful when you change parameters from `-h`.

Easy Workflows in MMseqs2

MMseqs2 includes easy workflows like `easy-search`, `easy-cluster`, and `easy-linclust`, which are designed to simplify common tasks like searching and clustering using FASTA/FASTQ file formats.

Easy-Search

The `easy-search` workflow enables searching with a FASTA/FASTQ file against another FASTA/FASTQ file or a pre-built MMseqs2 target database.

```
mmseqs easy-search examples/QUERY.fasta examples/DB.fasta alnResult.m8 tmp
```

In this command, `examples/QUERY.fasta` is the query file, `examples/DB.fasta` is the target database (either FASTA/FASTQ file or MMseqs2 database), `alnResult.m8` is the file for alignment results, and `tmp` is a temporary directory for intermediate files.

Easy-Cluster

`easy-cluster` clusters entries from a FASTA/FASTQ file using the cascaded clustering algorithm.

```
mmseqs easy-cluster examples/DB.fasta clusterRes tmp
```

Here, `examples/DB.fasta` is the input file, `clusterRes` is the output, and `tmp` is for temporary files.

Easy-Linclust

For larger datasets, `easy-linclust` offers an efficient clustering workflow, scaling linearly with input size.

```
mmseqs easy-linclust examples/DB.fasta clusterRes tmp
```

Similar to `easy-cluster`, but more suitable for handling very large datasets efficiently.

Using MMseqs2 Workflows and Modules

While these easy workflows simplify tasks by directly working with common file formats like FASTA/FASTQ, MMseqs2 also offers a wide range of modules for more specialized operations

like data transformation, filtering, and external program execution. These modules typically work with MMseqs2's internal database formats. For better performance and flexibility, it's often recommended to use MMseqs2's workflows and modules directly, especially for complex or large-scale bioinformatics analyses.

Downloading databases

Finding and setting up databases for different use cases can be a time-consuming step. To aid you in setting up databases for homology searches on protein, nucleotide and profile databases and for taxonomic annotation we provide the `databases` module. Running `mmseqs databases` without any parameters will show a list of databases we prepared (add `-h` for extended descriptions and references for each database):

```
# mmseqs databases
Usage: mmseqs databases <name> <o:sequenceDB> <tmpDir> [options]

  Name                    Type        Taxonomy    Url
- UniRef100               Aminoacid        yes    https://www.uniprot.org/help/uniref
- UniRef90                Aminoacid        yes    https://www.uniprot.org/help/uniref
- UniRef50                Aminoacid        yes    https://www.uniprot.org/help/uniref
- UniProtKB               Aminoacid        yes    https://www.uniprot.org/help/uniprotkb
- UniProtKB/TrEMBL        Aminoacid        yes    https://www.uniprot.org/help/uniprotkb
- UniProtKB/Swiss-Prot    Aminoacid        yes    https://uniprot.org
- NR                      Aminoacid        yes
↪ https://ftp.ncbi.nlm.nih.gov/blast/db/FASTA
- NT                      Nucleotide         -
↪ https://ftp.ncbi.nlm.nih.gov/blast/db/FASTA
- GTDB                    Aminoacid        yes    https://gtdb.ecogenomic.org
- PDB                     Aminoacid          -    https://www.rcsb.org
- PDB70                   Profile            -    https://github.com/soedinglab/hh-suite
- Pfam-A.full             Profile            -    https://pfam.xfam.org
- Pfam-A.seed             Profile            -    https://pfam.xfam.org
- Pfam-B                  Profile            -
↪ https://xfam.wordpress.com/2020/06/30/a-new-pfam-b-is-released
- CDD                     Profile                -
↪ https://www.ncbi.nlm.nih.gov/Structure/cdd/cdd.shtml
- eggNOG                  Profile            -    http://eggnog5.embl.de
- VOGDB                   Profile                -        https://vogdb.org
- dbCAN2                  Profile            -    http://bcb.unl.edu/dbCAN2
- SILVA                   Nucleotide           yes        https://www.arb-silva.de
- Resfinder               Nucleotide         -
↪ https://cge.cbs.dtu.dk/services/ResFinder
```

```
- Kalamari              Nucleotide      yes    https://github.com/lskatz/Kalamari
```

For example, run the following to download and setup the Swiss-Prot at the output path `outpath/swissprot`:

```
mmseqs databases UniProtKB/Swiss-Prot outpath/swissprot tmp
```

In this case, since Swiss-Prot has a value yes in the `Taxonomy` column above, all necessary files to use it as a valid seqTaxDB will be downloaded and prepared by the `databases` command.

The `databases` workflow will further more create a file with the `.version` suffix that indicates version number or - if not available - the date of the download.

Please open a GitHub issue to request additional databases.

Searching

Before searching, you need to convert your FASTA file containing query sequences and target sequences into a sequence DB. You can use the query database `examples/QUERY.fasta` and target database `examples/DB.fasta` to test the search workflow:

```
mmseqs createdb examples/QUERY.fasta queryDB
mmseqs createdb examples/DB.fasta targetDB
```

These calls should generate five files each, e.g. `queryDB`, `queryDB_h` and its corresponding index file `queryDB.index`, `queryDB_h.index` and `queryDB.lookup` from the FASTA `QUERY.fasta` input sequences.

The `queryDB` and `queryDB.index` files contain the amino acid sequences, while the `queryDB_h` and `queryDB_h.index` file contain the FASTA headers. The `queryDB.lookup` file contains a list of tab separated fields that map from the internal identifier to the FASTA identifiers.

For the next step, an index file of the `targetDB` is computed for a fast read-in. It is recommended to compute the index if the `targetDB` is reused for several searches. If only few searches against this database will be done, this step should be skipped.

```
mmseqs createindex targetDB tmp
```

This call will create a `targetDB.idx` file. It is just possible to have one index per database.

Then generate a directory for temporary files. MMseqs2 can produce a high IO on the file system. It is recommended to create this temporary folder on a local drive.

```
mkdir tmp
```

Please ensure that in case of large input databases tmp provides enough free space. For disk space requirements, see the section Disk Space.

The alignment consists of two steps the prefilter and alignment. To run the search, type:

```
mmseqs search queryDB targetDB resultDB tmp
```

Search as standard does compute the score only. If you need the alignment information add the option "-a". The speed and sensitivity of the search can be adjusted with -s parameter and should be adapted based on your use case (see setting sensitivity -s parameter). A very fast search would use a sensitivity of -s 1.0, while a very sensitive search would use a sensitivity of up to -s 7.0. A detailed guide how to speed up searches is here.

The output can be customized with the --format-output option e.g. --format-output "query,target,qaln,taln" returns the query and target accession and the pairwise alignments in tab separated format. You can choose many different output columns.

Then, convert the result database into a BLAST tab formatted file (option -m 8 in legacy blast, -outfmt 6 in blast+):

```
mmseqs convertalis queryDB targetDB resultDB resultDB.m8
```

convertalis columns

The file is formatted as a tab-separated list with 12 columns: (1,2) identifiers for query and target sequences/profiles, (3) sequence identity, (4) alignment length, (5) number of mismatches, (6) number of gap openings, (7-8, 9-10) domain start and end-position in query and in target, (11) E-value, and (12) bit score.

Read more about searching here.

GPU-accelerated search

MMseqs2 has a GPU-accelerated search backend since release 16. GPU-accelerated searches parallelize computation within a single query rather than across multiple queries (default of MMseqs2 CPU), enabling very fast searches even for small protein sets. In contrast to the k-mer based search, this mode does NOT allow to trade-off sensitivity for speed by reducing the search sensitivity -s. It always operates at maximum sensitivity. To utilize GPU acceleration, you need for format the target database for GPU use with the makepaddedseqdb module and pass the --gpu 1 parameter to search or easy-search. Clustering does not support GPUs yet.

```
mmseqs createdb examples/DB.fasta targetDB
mmseqs makepaddedseqdb targetDB targetDB_gpu
```

```
mmseqs rmdb targetDB
mmseqs easy-search examples/QUERY.fasta targetDB_gpu resultDB tmp --gpu 1
```

To enable real-time searches against a database with minimal overhead (particularly important in server-like environments), GPU-compatible databases can be indexed using `createindex`. The `--index-subset 2` parameter is useful for omitting the large k-mer data structures required by the default MMseqs2 k-mer-based search, reducing memory usage to approximately 1 byte per residue in the target database instead of 7 bytes for the full k-mer index. However, this index will only work for GPU-based searches or CPU-based searches with the `ungappedprefilter`, which is also accessed in `search`/`easy-search` through `--prefilter-mode 1` or 3. To additionally reduce process spawning overhead, the GPU database can be held resident on GPU(s) through the `gpuserver` module.

```
mmseqs createindex targetDB_gpu tmp --index-subset 2
# spawn in background
mmseqs gpuserver targetDB_gpu &
PID=$!
mmseqs search queryDB targetDB_gpu resultDB tmp --gpu 1 --gpu-server 1 --db-load-mode
 ↪ 2
# stop the GPU server again
kill $PID
```

> [!NOTE] The `gpuserver` as well as `search` require to use the same `--max-seqs` and `--prefilter-mode` parameter as well as the same `CUDA_VISIBLE_DEVICES`.

Multi-GPU usage

If present, MMseqs2-GPU can utilize multiple GPUs. It spreads the target database evenly across multiple GPUs to accelerate the search further. We do not expose a special parameter for this, instead you should use the `CUDA_VISIBLE_DEVICES` environment variable to chose which GPUs MMseqs2 accesses.

```
CUDA_VISIBLE_DEVICES=0,1 mmseqs easy-search examples/QUERY.fasta targetDB_gpu resultDB
 ↪ tmp --gpu 1
```

Database larger than GPU memory

MMseqs2-GPU efficiently processes databases that exceed GPU memory by streaming data directly from host RAM using asynchronous CUDA streams. If the database fits into host RAM but exceeds GPU memory, MMseqs2-GPU maintains approximately 60% of its peak performance.

Clustering

Before clustering, convert your FASTA database into the MMseqs2 database (DB) format:

```
mmseqs createdb examples/DB.fasta DB
```

You will need to specify a directory for temporary files. Please ensure that in case of large input databases `tmp` provides enough free space. For the disk space requirements, see the section Disk space.

Run the clustering of your database `DB` by executing the following command. MMseqs2 will return the result database files `DB_clu`, `DB_clu.index`:

```
mmseqs cluster DB DB_clu tmp
```

To generate a TSV formatted output file from the output file, type:

```
mmseqs createtsv DB DB DB_clu DB_clu.tsv
```

You can adjust the sequence identity threshold with `--min-seq-id` and the alignment coverage with `-c` and `--cov-mode` (See How to set the right alignment coverage to cluster). MMseqs2 will set the sensitivity parameters automatic based on target sequence identity (`--min-seq-id`), if it is not already specified through the `-s` or `--k-score` parameters.

Sequence information can be added by using createseqfiledb and result2flat can produce a result.

```
mmseqs createseqfiledb DB DB_clu DB_clu_seq
mmseqs result2flat DB DB DB_clu_seq DB_clu_seq.fasta
```

Read more about clustering here.

Linclust

Linclust is a clustering in linear time. It is magnitudes faster but a bit less sensitive than clustering.

Before clustering, convert your FASTA database into the MMseqs2 database (DB) format:

```
mmseqs createdb examples/DB.fasta DB
```

To run linclust to generate a clustering of your database `DB` execute the following command. The result database follows the same format as the clustering format:

```
mmseqs linclust DB DB_clu tmp
```

To extract the representative sequences from the clustering result call:

```
mmseqs createsubdb DB_clu DB DB_clu_rep
mmseqs convert2fasta DB_clu_rep DB_clu_rep.fasta
```

Updating a clustered database

It is possible to update previous clustered databases without re-clustering everything from the scratch.

Let us create an older version of the `DB.fasta` (in the example folder) by removing 1000 sequences:

```
awk '/^>/{seqCount++;} {if (seqCount <= 19000) {print $0;}}' DB.fasta >
 ↪  DB_trimmed.fasta
```

Now we create the sequence database of this simulated old sequence and the corresponding clustering:

```
mmseqs createdb DB_trimmed.fasta DB_trimmed
mmseqs cluster DB_trimmed DB_trimmed_clu tmp
```

To update the clustering `DB_trimmed_clu` with the new version of your database `DB_new`:

```
mmseqs createdb DB.fasta DB_new
mmseqs clusterupdate DB_trimmed DB_new DB_trimmed_clu DB_new_updated DB_update_clu tmp
```

`DB_update_clu` contains now the freshly updated clustering of DB_new. Furthermore, the `clusterupdate` creates a new sequence database `DB_new_updated` that has consistent identifiers with the previous version. Meaning, the same sequences in both sets will have the same numeric identifier. All modules afterwards (for example `convertalis`) expect this sequence database to be passed.

Read more about the cluster updating here.

Overview of folders in MMseqs2

- `bin`: `mmseqs`
- `data`: BLOSUM matrices and the workflow scripts (`blastp.sh`, `blastpgp.sh`, `cascaded_clustering.sh`, `linclust.sh`, `searchtargetprofile.sh`, `clustering.sh`)
- `examples`: test data `QUERY.fasta` and `DB.fasta`
- `util`: Contains the Bash parameter completion script.

Overview of important MMseqs2 Modules

MMseqs2 contains five workflows that combine the core MMseqs2 modules (prefilter, align, kmermatcher, rescorediagonal and clust) and several other smaller ones.

Workflows:

- `mmseqs search`: Compares all sequences in the query database with all sequences in the target database, using the prefiltering and alignment modules. MMseqs2 search supports sequence/sequence, profile/sequence or sequence/profile searches.
- `mmseqs cluster`: Clusters sequences by similarity. It compares all sequences in the sequence DB with each other using mmseqs search, filters alignments according to user-specified criteria (max. E-value, min. coverage,...), and runs mmseqs clust to group similar sequences together into clusters.
- `mmseqs linclust`: Clusters sequences by similarity in linear time. It clusters magnitudes faster than `mmseqs cluster` but is less sensitive.
- `mmseqs clusterupdate`: MMseqs2 incrementally updates a clustering, given an existing clustering of a sequence database and a new version of this sequence database (with new sequences being added and others having been deleted).
- `mmseqs taxonomy` Taxonomy assignment by computing the lowest common ancestor of homologs using 2bLCA.
- `mmseqs map`: Map calls the search workflow with different default parameters and alignment strategy. Map should be used for high identities searches, which is more of a technical task rather than measuring homology.

And the five core modules:

- `mmseqs prefilter`: Computes k-mer similarity scores between all sequences in the query database and all sequences in the target database.
- `mmseqs kmermatcher`: finds exact k-mer matches between all input sequences in linear time.
- `mmseqs align`: Computes Smith-Waterman alignment scores between all sequences in the query database and the sequences of the target database whose prefiltering scores computed by `mmseqs prefilter` pass a minimum threshold.
- `mmseqs rescorediagonal`: Computes 1D optimal score between all sequences in the query database and the sequences of the target database, which passed the prefilter `mmseqs prefilter` or `mmseqs kmermatcher`.
- `mmseqs clust`: Computes a similarity clustering of a sequence database based on Smith Waterman alignment scores of the sequence pairs computed by `mmseqs align`.

MMseqs2 has more than 96 modules in total. We provide modules for clustering, searching, alignments, taxonomy, and data transformation. For a complete list of all available modules, execute `mmseqs` without arguments.

Description of workflows

MMseqs2 workflows combine modules in shell scripts. The executed script can be found in the respective temporary directory.

Batch sequence searching using `mmseqs search`

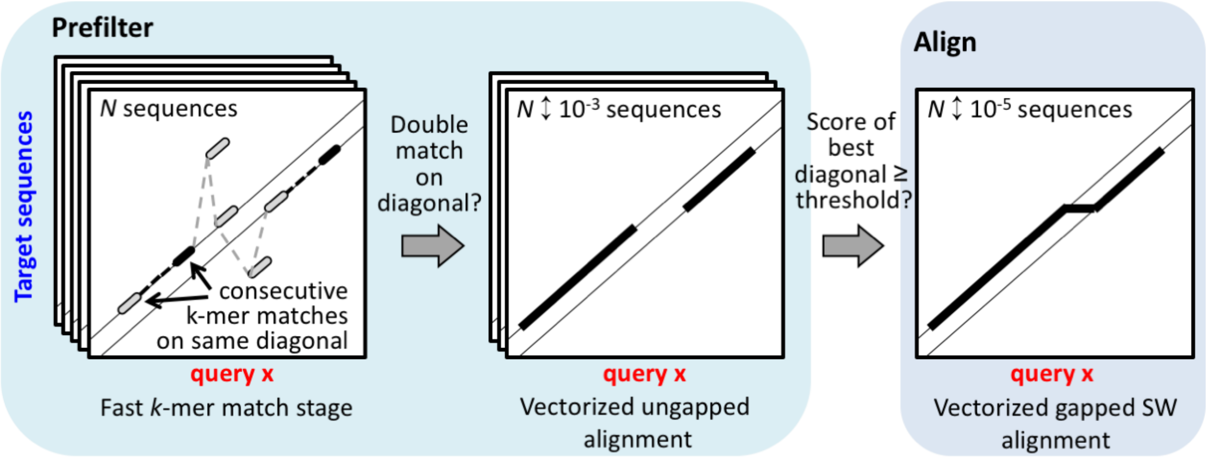

Figure 0.1: Search workflow

For searching a database, query and target database have to be converted by `createdb` in order to use them in MMseqs2. The search can be executed by typing:

```
mmseqs search queryDB targetDB outDB tmp
```

MMseqs2 supports iterative searches which are similar to PSI-BLAST. The following program call will run two iterations through the database. In the first iteration sequences are searched against sequence and in the second one profiles are used to search against sequences.

MMseqs2 will use the output for the first iteration sequence-sequence search to computes a profile (result2profile). The profile will be used as input in the next search iteration. Accepted hits are aligned only once and removed from later iterations.

```
mmseqs search queryDB targetDB outDB tmp --num-iterations 2
```

This workflow combines the prefiltering and alignment modules into a fast and sensitive batch sequence search that compares all sequences in the query database with all sequences in the target database.

Query and target databases may be identical. The program outputs for each query sequence all database sequences satisfying the search criteria (such as sensitivity).

MMseqs2 can precompute the prefilter index `createindex` to speed up subsequent prefilter index read-in. We recommend using an index for iterative searches or if a target database will be reused several times. However reading the index can be bottle neck when using a network file systems (NFS). It is recommended to keep the index on a local hard drive. If storing the index file on a local hard drive is not possible and the NFS is a bottleneck than there are two solutions:

1. Load the index into main memory using the module `touchdb` or `vmtouch` (https://github.com/hoytech/vmtouc[illegible] MMseqs2 can be forced to use the main memory database by using the parameter `--db-load-mode 2`.
2. Do not create an index, MMseqs2 will compute an index on the fly this reduces the IO volume by roughly a factor of seven.

More details to the underlying search algorithm can be found in the section Computation of prefiltering scores using mmseqs prefilter, and the important parameter list can be found in section Search workflow.

Expanded cluster searches

```
This section is work in progress. Expandable searches will be made more easy to setup
↪  and to use.
```

To better handle the rapidly increasing size of protein databases, we introduce a cluster-centroid search mode that then expands the centroid search hits to the respective cluster members.

Downloading precomputed expandable profile databases

Download one of the three provided expandable profile databases from https://colabfold.mmseqs.com and convert it to the MMseqs2 databases format with `tsv2exprofiledb`:

```
wget http://wwwuser.gwdg.de/~compbiol/colabfold/uniref30_2103.tar.gz
tar xzvf uniref30_2103.tar.gz
mmseqs tsv2exprofiledb uniref30_2103 uniref30_2103_db
```

This set of databases consists of four databases: * `<db>` The centroid sequences. In the provided ColabFold databases, these are cluster consensus sequences. * `<db>_seq <db>_h/<db>_seq_h` Sequence and header databases for all cluster member sequences. * `<db>_aln` Alignment database of cluster centroids to the respective cluster members.

Searching against expandable profile databases

The `expandaln` module expands for each centroid hit the cluster members. Here, we add an additional realignment step to increase alignment quality. `expandaln` doesn't perform alignments and

projects the alignment of query to member sequences based on the alignment of centroid to member sequence (details in the upcoming manuscript).

```
mmseqs search queryDB uniref30_2103_db res tmp -a
mmseqs expandaln queryDB uniref30_2103_db res uniref30_2103_db_aln res_expanded
mmseqs align queryDB uniref30_2103_db_seq res_expanded res_expanded_realign
mmseqs convertalis queryDB uniref30_2103_db_seq res_expanded res_expanded_realign.m8
```

`res_expanded_realign.m8` will contain the BLAST-tab formatted alignment results (see Searching section).

Sensitivity of the centroid search can be increased with the usual parameters (e.g. `-s` and `--num-iterations`).

Using a precomputed index with expandable profile databases

A precomputed index can be created to enable many rapid searches against an expandable profile database.

```
mmseqs tsv2exprofiledb uniref30_2103 uniref30_2103_db
mmseqs createindex uniref30_2103_db tmp --split 1
```

A precomputed index is only helpful if enough system RAM is available to keep the created index file fully resident in RAM. You can ensure that the precomputed index is fully in RAM with e.g. vmtouch (https://github.com/hoytech/vmtouch):

```
sudo vmtouch -t -l -d -w uniref30_2103_db.idx
```

`uniref30_2103_db.idx` will contain all the databases mentioned above and can be searched as follows:

```
mmseqs search queryDB uniref30_2103_db res tmp --db-load-mode 2
mmseqs expandaln queryDB uniref30_2103_db.idx res uniref30_2103_db.idx res_expanded
 ↪  --db-load-mode 2
mmseqs align queryDB uniref30_2103_db.idx res_expanded res_expanded_realign
 ↪  --db-load-mode 2
mmseqs convertalis queryDB uniref30_2103_db.idx res_expanded res_expanded_realign.m8
 ↪  --db-load-mode 2
```

Translated sequence searching

The search workflow can perform translated searches with nucleotide databases on either query or target, or both sides. It will trigger a search similar to BLASTX, TBLASTN and TBLASTX respectively. The search uses the `extractorfs` module to detect all open reading frames (ORFs)

on all six frames and translates them into proteins. Per default, MMseqs2 extracts all ORFs per frame starting from any codon until a stop codon occurs (`--orf-start-mode 1`) that are longer than 30 amino acid residues (`--min-length 30`). All ORFs are translated by the `translatenucs` module using the canonical translation table (`--translation-table 1`). After the protein-protein search all alignments will be offset to the original nucleotide corrdinates by the `offsetalignment` module.

To perform a translated search, first prepare your database using `createdb`. It can automatically detect if the input are amino acids or nucleotides.

```
mmseqs createdb ecoli.fna ecoli_genome
mmseqs createdb ecoli.faa ecoli_proteins
```

A nucleotide/protein (BLASTX) search can be triggered using the nucleotide database on the query database side.

```
mmseqs search ecoli_genome ecoli_proteins alnDB tmp
```

A protein/nucleotide (TBLASTN) search can be triggered using the nucleotide database on the target database side.

```
mmseqs search ecoli_proteins ecoli_genome alnDB tmp
```

A translated nucleotide/nucleotide (TBLASTX) search can be trigged using the flag `--search-type 2`

```
mmseqs search genome_orfs_aa ecoli_genome alnDB tmp --search-type 2
```

Mapping very similar sequences using `mmseqs map`

The `map` workflow of MMseqs2 finds very similar sequence matches in a sequence database. First it calls the prefilter module (with a low sensitivity setting) to detect high scoring diagonals and then computes an ungapped alignment with the rescorediagonal module. In contrast to the normal search, for maximum speed no gapped alignment is computed, query sequences are not masked for low complexity regions and no compositional bias correction is applied.

```
mmseqs map queryDB targetDB resultDB tmp
```

MMseqs2 will provide a sorted (by E-value) list of best matches in `resultDB`. The best hit can be extracted with:

```
mmseqs filterdb resultDB bestResultDB --extract-lines 1
```

The format of `resultDB` is the same as in alignment format of the normal `search` workflow. The mapping workflow can also be used in iterative-best-hit mode, where each query that does not find any match is searched with higher sensitivity again.

If either `queryDB` or `targetDB` is a nucleotide sequence database, MMseqs2 will use the translated sequence search mode described above.

Clustering databases using `mmseqs cluster` or `mmseqs linclust`

To cluster a database, MMseqs2 needs a sequence database converted with `createdb` and an empty directory for temporary files. Then, you can run the cascaded clustering with:

```
mmseqs cluster inDB outDB tmp
```

The sensitivity of the clustering can be adjusted with the `-s` option. MMseqs2 will automatically adjust the sensitivity based on the `--min-seq-id` parameter, if `-s` is not provided.

Linclust can be used by calling `linclust`. The sensitivity can be adjusted by `--kmer-per-seq` (default 20).

```
mmseqs linclust inDB outDB tmp
```

The clustering workflow `cluster` combines the prefiltering, alignment and clustering modules into either a simple clustering or a cascaded clustering of a sequence database. There are two ways to execute the clustering:

- The *Simple clustering* `--single-step-clustering` runs the hashclust and prefiltering, alignment and clustering modules with predefined parameters with a single iteration.
- *Cascaded clustering* (default) clusters the sequence database using the as first step linclust and then prefiltering, alignment and clustering modules incrementally in three steps.

Clustering criteria

MMseqs2/Linclust and Linclust has three main criteria, inferred by a local alignment, to link two sequences by an edge:

(1) a maximum E-value threshold (option `-e [0,\infty[`) computed according to the gap-corrected Karlin-Altschul statistics using the ALP library.

(2) a minimum coverage (option `-c [0,1]`, which is defined by the number of aligned residue pairs divided by either the maximum of the length of query/centre and target/non-centre sequences `alnRes/max(qLen,tLen)` (default mode, `--cov-mode 0`), or by the length of the target/non-centre sequence `alnRes/tLen` (`--cov-mode 1`), or by the length of the

query/centre `alnRes/qLen` (`--cov-mode 2`). Read more about how coverage is computed in section How to set the right alignment coverage to cluster.

(3) a minimum sequence identity (`--min-seq-id` [0,1]) with option `--alignment-mode 3` defined as the number of identical aligned residues divided by the number of aligned columns including internal gap columns, or, by default, defined by a highly correlated measure, the equivalent similarity score of the local alignment (including gap penalties) divided by the maximum of the lengths of the two locally aligned sequence segments. The score per residue equivalent to a certain sequence identity is obtained by a linear regression using thousands of local alignments as training set.

Cascaded clustering

The cascaded clustering workflow first runs `linclust`, our linear-time clustering module, that can produce clustering's down to 50% sequence identity in very short time.

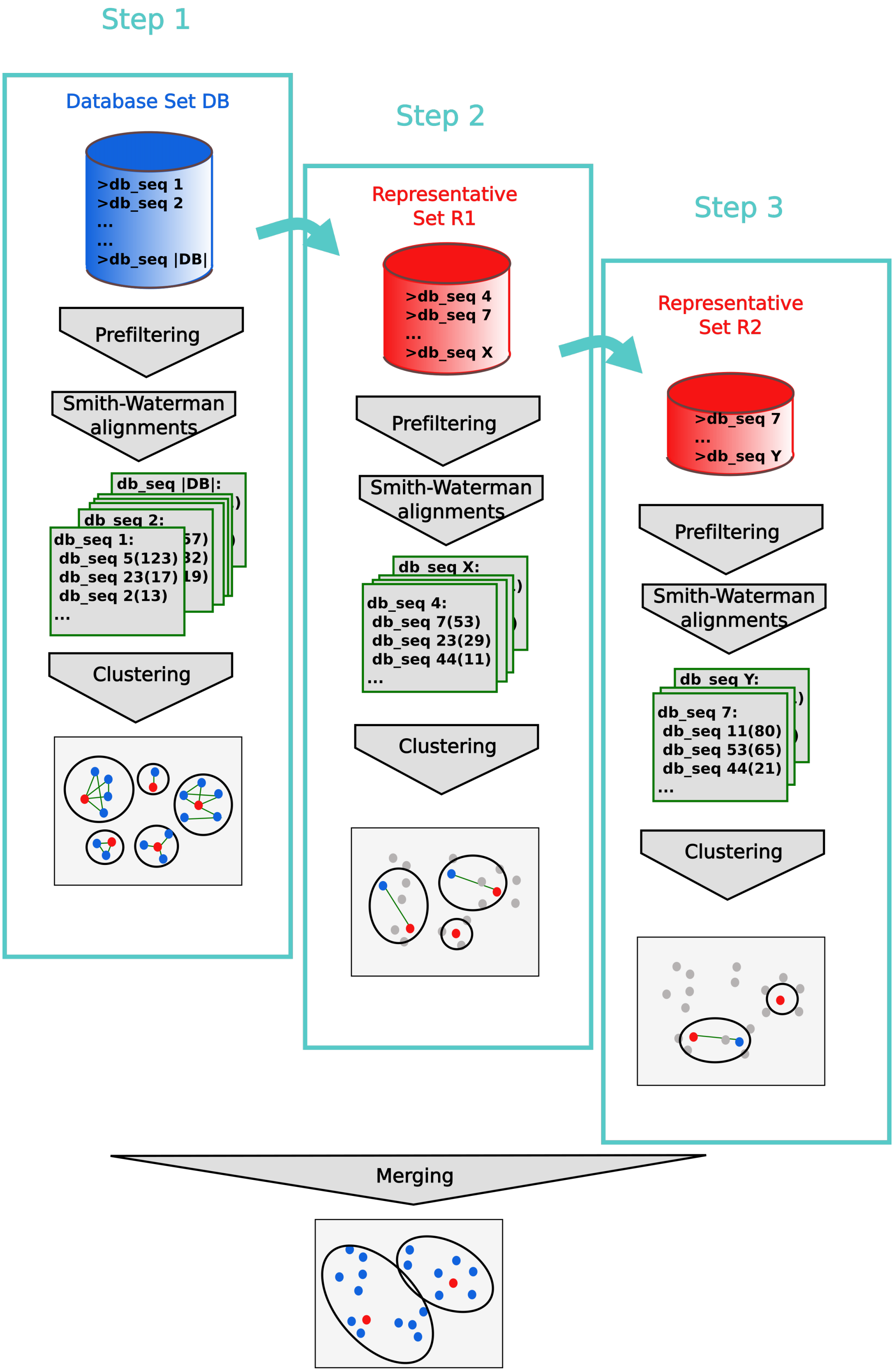

Figure 0.2: Cascaded clustering

To achieve lower sequence identities and/or to further improve the resulting clusters, we continue with three cascaded clustering steps: In the first step of the cascaded clustering the prefiltering runs with a low sensitivity of 1 and a very high result significance threshold, in order to accelerate the calculation and search only for hits with a very high sequence identity. Then alignments are calculated and the database is clustered. The second step takes the representative sequences of the first clustering step and repeats the prefiltering, alignment and clustering steps. This time, the prefiltering is executed with a higher sensitivity and a lower result significance threshold for catching sequence pairs with lower sequence identity. In the last step, the whole process is repeated again with the final target sensitivity. At last, the clustering results are merged and the resulting clustering is written to the output database.

Cascaded clustering is performed in default because of its increase in speed. Also, it allows very large cluster sizes in the end clustering resulting from cluster merging (note that cluster size can grow exponentially in the cascaded clustering workflow), which is not possible with the simple clustering workflow because of the limited maximum number of sequences passing the prefiltering and the alignment. Computing an exhaustive all against all distance matrix would take too much hard-disk. However it is possible to perform a single step clustering using `--single-step-clustering`.

Cascaded clustering has many advantages but comes with one caveat. Since the representative of a cluster can change with every iteration it can happen that some members that were already close to a clustering do not fulfill the clustering criteria anymore. However, we can correct this by reassigning the sequences. To activate the reassignment use `--cluster-reassign`. This mode removes sequences from the cascaded cluster result that do not fulfill the cluster criteria and reassigns them (if possible) to a different cluster.

Clustering modes

All clustering modes transform the alignment results into an undirected graph. In this graph notation, each vertex (i.e. node) represents a sequence, which is connected to other sequences by edges. An edge between a pair of sequences is introduced if the alignment criteria (e.g. `--min-seq-id`, `-c` and `-e`) are fulfilled.

The Greedy Set cover (`--cluster-mode 0`) algorithm is an approximation for the NP-complete optimization problem called set cover.

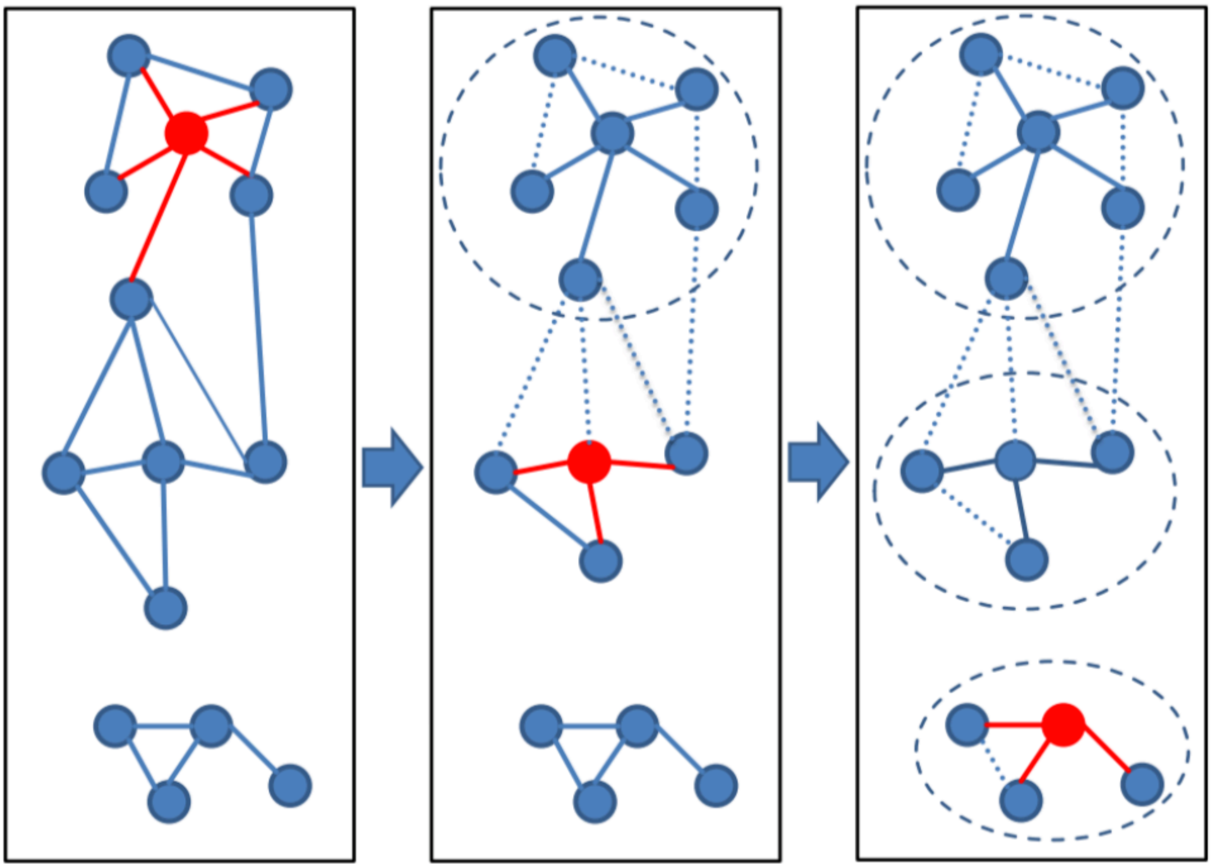

Figure 0.3: Set Cover clustering

Greedy set cover works by iteratively selecting the node with most connections and all its connected nodes to form a cluster and repeating until all nodes are in a cluster. The greedy set cover is followed by a reassignment step. A Cluster member is assigned to another cluster centroid if their alignment score was higher.

Connected component (`--cluster-mode 1`) uses transitive connection to cover more remote homologs.

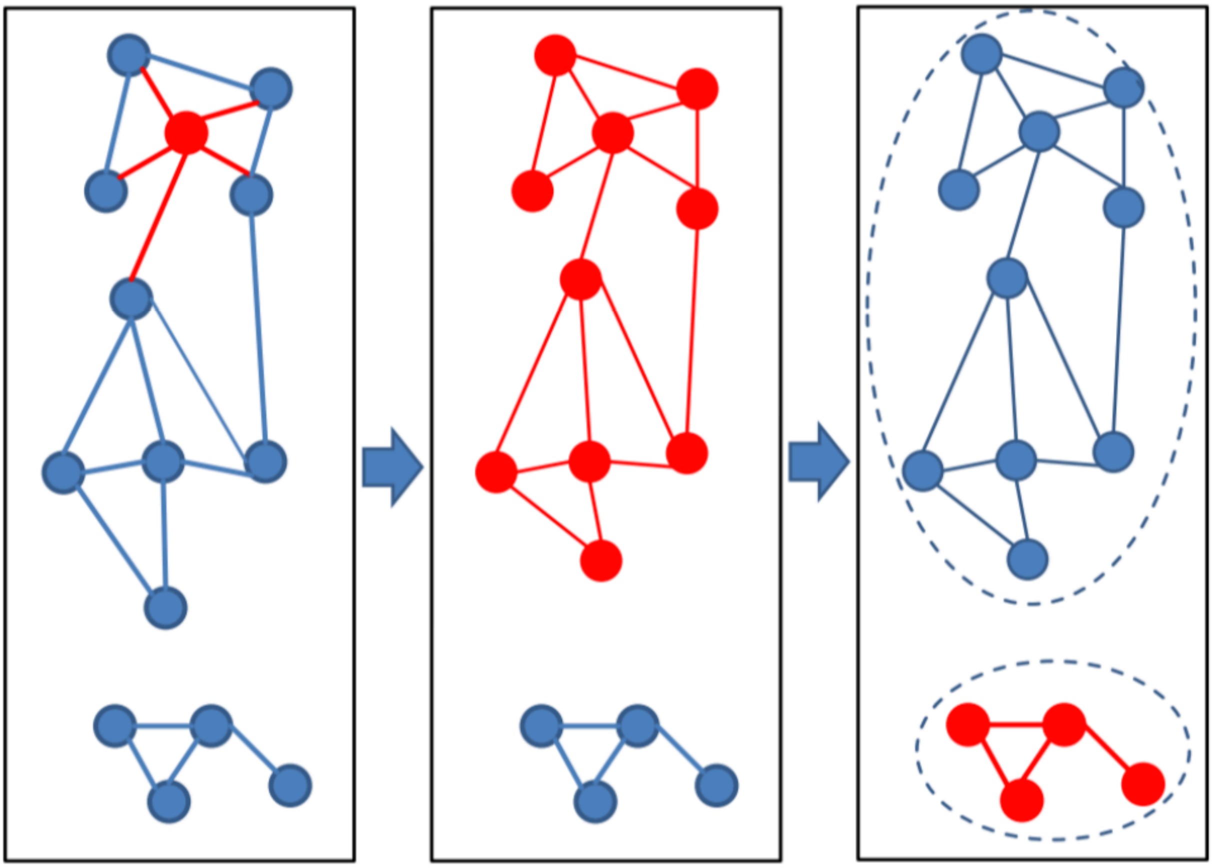

Figure 0.4: Connected component clustering

In connected component clustering starting at the mostly connected vertex, all vertices that are reachable in a breadth-first search are members of the cluster.

Greedy incremental (`--cluster-mode 2`) works analogous to CD-HIT clustering algorithm.

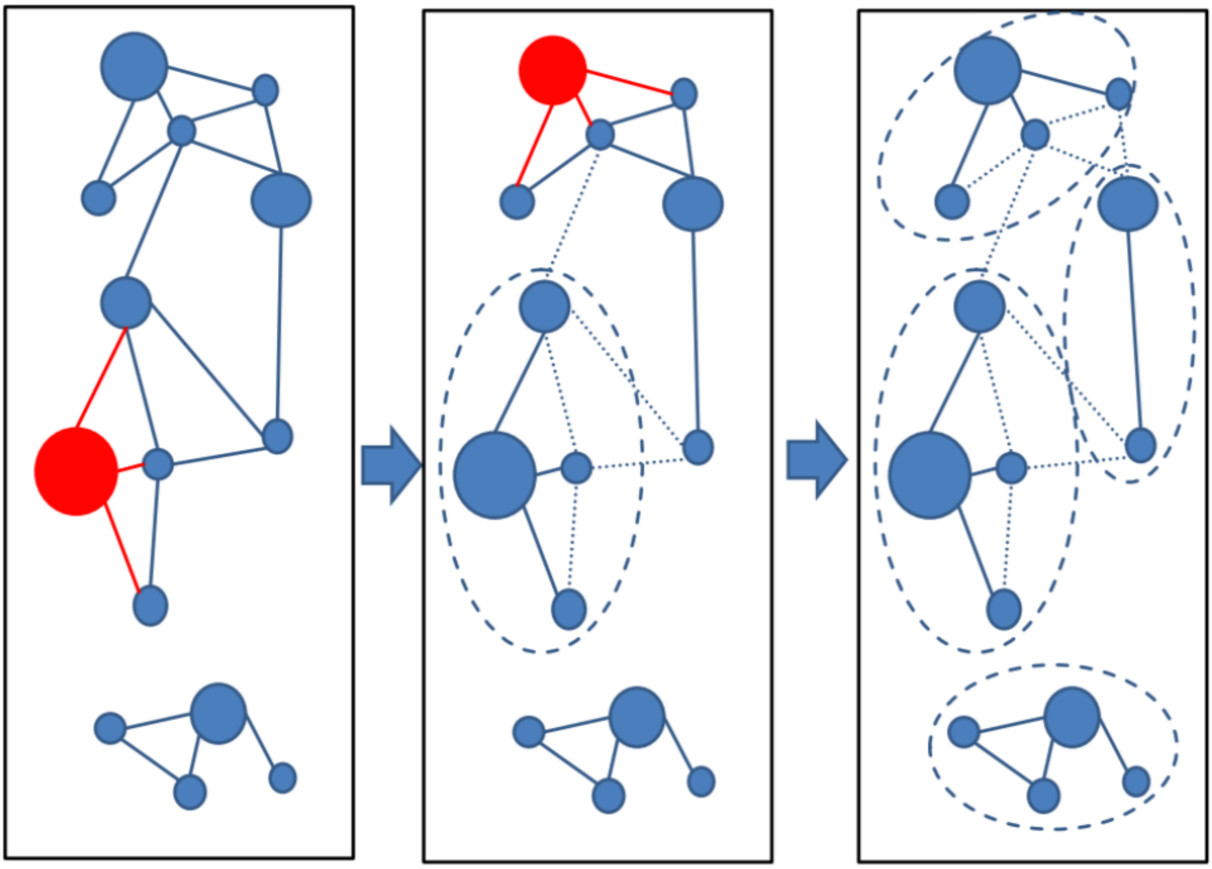

Figure 0.5: Greedy incremental clustering

Greedy incremental clustering takes the longest sequence (indicated by the size of the node) and puts all connected sequences in that cluster, then repeatedly the longest sequence of the remaining set forms the next cluster.

Linear time clustering using `mmseqs linclust`

Linclust can cluster sequences down to 50% pairwise sequence similarity and its runtime scales linearly with the input set size.

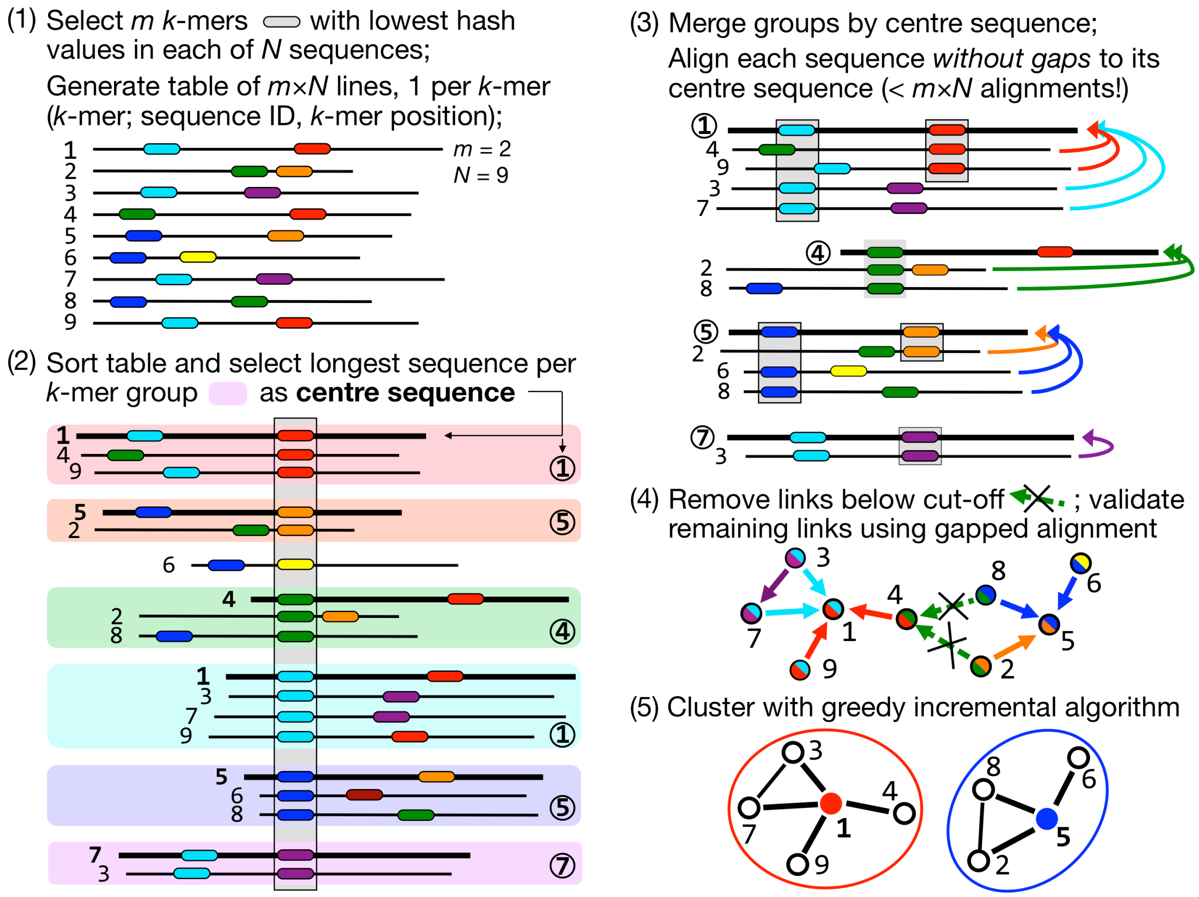

Figure 0.6: Linclust algorithm

Linear-time clustering algorithm. Steps 1 and 2 find exact k-mer matches between the N input sequences that are extended in step 3 and 4.

(1) Linclust selects in each sequence the `m` (default: 20) k-mers with the lowest hash function values, as this tends to select the same k-mers across homologous sequences. It uses a reduced alphabet of 13 letters for the k-mers and sets k=10 for sequence identity thresholds below 90% and k=14 above. It generates a table in which each of the mN lines consists of the k-mer, the sequence identifier, and the position of the k-mer in the sequence.

(2) Linclust sorts the table by k-mer in quasi-linear time, which identifies groups of sequences sharing the same k-mer (large shaded boxes). For each k-mer group, it selects the longest sequence as centre. It thereby tends to select the same sequences as centre among groups sharing sequences.

(3) It merges k-mer groups with the same centre sequence together: red + cyan and orange + blue and compares each group member to the centre sequence in two steps: by global Hamming

distance and by gapless local alignment extending the k-mer match.

(4) Sequences above a score cut-off in step 3 are aligned to their centre sequence using gapped local sequence alignment. Sequence pairs that satisfy the clustering criteria (e.g. on the E-value, sequence similarity, and sequence coverage) are linked by an edge.

(5) The greedy incremental algorithm finds a clustering such that each input sequence has an edge to its cluster's representative sequence. Note that the number of sequence pairs compared in steps 3 and 4 is less than mN, resulting in a linear time complexity.

Run Linclust

Linclust needs a sequence database created by `createdb` and an empty directory for temporary files. Then, you can run the clustering with the following command:

```
mmseqs linclust inDB outDB tmp
```

Increasing the k-mers selected per sequence increases the sensitivity of linclust at a moderate loss of speed. Use the parameter `--kmer-per-seq` to set the number of k-mers selected per sequence. More k-mers per sequences results in a higher sensitivity.

The output format of linclust is the same format as in `mmseqs cluster`. See section Clustering Format.

Updating a clustering database using `mmseqs clusterupdate`

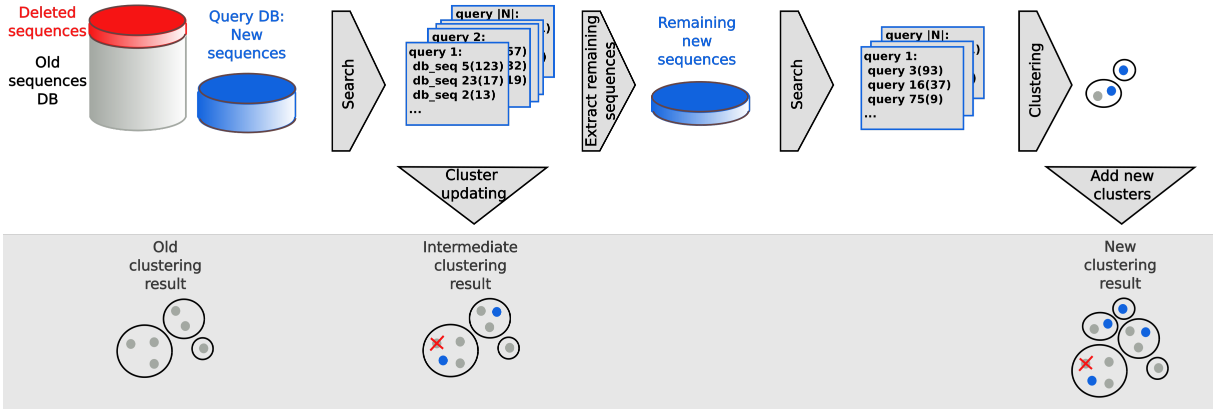

Figure 0.7: Update clustering

To run the updating, you need the old and the new version of your sequence database in sequence db format, the clustering of the old database version and a directory for the temporary files:

```
mmseqs clusterupdate oldDB newDB cluDB_old newDB_updated cluDB_updated tmp
```

This workflow efficiently updates the clustering of a database by adding new and removing outdated sequences. It takes as input the older sequence database, the corresponding clustering, and the new version of the sequence database. Then it adds the new sequences to the clustering and removes the sequences that were removed in the new database. Sequences which are not similar enough to any existing cluster will be representatives of new clusters.

`clusterupdate` creates a new sequence database `newDB_updated` that has consistent identifiers with the previous sequence databases. Meaning, the same sequences in both sets will have the same numeric identifier. All modules afterwards (for example `convertalis`) expect this sequence database to be passed.

Taxonomy assignment

Terminology

An MMseqs2 database `seqTaxDB` is a sequence database augmented with taxonomic information and a mapping file from each database key to its taxon id. Such a database includes the following files: `seqTaxDB`, `seqTaxDB.index`, `seqTaxDB.dbtype`, `seqTaxDB.lookup`, `seqTaxDB_h`, `seqTaxDB_h.index`, `seqTaxDB_h.dbtype`, `seqTaxDB_mapping` and either the taxonomy flat file databases `seqTaxDB_nodes.dmp`, `seqTaxDB_names.dmp`, `seqTaxDB_merged.dmp` or `seqTaxDB_taxonomy` a binary version of the former files (created by `createtaxdb` which reduces the read-in time of the taxonomy database).

As detailed in the following sections, there are several ways to create and manipulate a `seqTaxDB`. Once created, `seqTaxDB` can be used by various MMseqs2 modules to assign taxonomic labels to sequences based on their similarities to the sequences of `seqTaxDB`. The result database of searching sequences against a `seqTaxDB` is referred to as `taxonomyResult`.

Creating a seqTaxDB

The `databases` module provides a easy way to download and setup taxonomy databases that we predefined (see Downloading databases). Follow this section to learn how to manually create a seqTaxDB.

First, create a sequence database:

```
mmseqs createdb seqTax.fasta seqTaxDB
```

Next, augment it with taxonomic information.

If your seqTaxDB **contains Uniprot ids**, the easiest way to do so is to call:

```
mmseqs createtaxdb seqTaxDB tmp
```

This module will download the Uniprot idmapping and ncbi-taxdump and map the identifier of the seqTaxDB to NCBI taxonomic identifier. By default, createtaxdb downloads the Uniprot id mapping file (idmapping.dat.gz), and thus only supports Uniprot identifiers.

If your seqTaxDB **does not contain Uniprot ids** or you wish to provide an alternative source for the taxonomic information, please follow the instructions here:

- To create a seqTaxDB from an existing NCBI BLAST database (such as nr or nt), see the section Create a seqTaxDB from an existing BLAST database.
- For the SILVA database use mmseqs databases or see Create a seqTaxDB for SILVA for an example how to build it yourself.
- For the GTDB database see Create a seqTaxDB for GTDB.
- For other database types, the mapping must be created manually, as described in section Create a seqTaxDB by manual annotation of a sequence database.

Filtering a seqTaxDB

Once you have a seqTaxDB, you can use filtertaxseqdb to retain (or exclude) sequences based on their taxonomic labels. For example, if you wish to retain only sequences of eukaryotic origin:

```
mmseqs filtertaxseqdb seqTaxDB seqTaxOnlyEuksDB --taxon-list 2759
```

or to exclude any human sequences:

```
mmseqs filtertaxseqdb seqTaxDB seqTaxNoHumanDB --taxon-list '!9606'
```

The concept of LCA

By identifying homologs through searches against a seqTaxDB, MMseqs2 can compute the lowest common ancestor. This lowest common ancestor is a robust taxonomic label for unknown sequences.

MMseqs2 assigns taxonomic labels based on an accelerated approximation (Supp. Material in Mirdita et al., 2021) of the 2bLCA protocol (Hingamp et al., 2013) --lca-mode 3 (default). However, we implemented several assignment strategies like the lowest common ancestor of all equal scoring top hits --lca-mode 4.

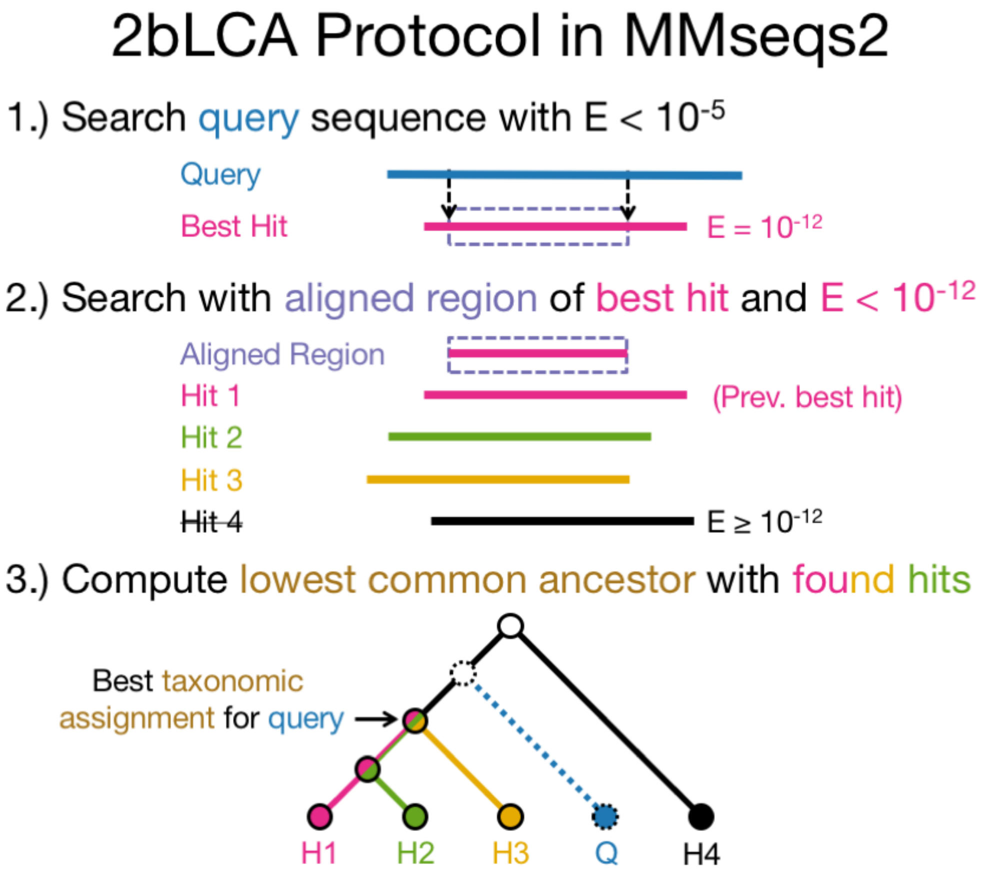

Figure 0.8: 2bLCA protcol (Hingamp et. al, 2013)

The second search can be disabled with `--lca-mode 1`. The LCA will then be only computed through the usual search workflow parameters (`--max-accept`, `-e`, etc.).

The LCA implementation is based on the Go implementation of blast2lca software on GitHub. It implements the LCA computation efficiently through *Range Minimum Queries* through a dynamic programming approach.

Using seqTaxDB for taxonomy assignment

The MMseqs2 module `taxonomy` calls an internal module `lca` that implements an LCA assignment for sequences by querying them against a seqTaxDB. You can control the information provided about the lineage by using the `tax-lineage` parameter. The default mode is `--tax-lineage 0` and does not include information about the ancestry of the assigned taxon. The mode `--tax-lineage 1` will add a column with the full lineage names, prefixed with their short rank (e.g., `-_cellular organisms;d_Eukaryota;...;g_Saccharomyces;s_Saccharomyces`

cerevisiae) and mode --tax-lineage 2 will add a column with the full lineage NCBI taxids (e.g., 131567;2759;...;4930;4932):

```
mmseqs taxonomy queryDB seqTaxDB taxonomyResult tmp
```

Taxonomy output and TSV

Depending on the input MMseqs2 taxonomy will provide different slightly different output, as different algorithms are used with varying context. For protein-protein, nucleotide-nucleotide, and protein-nucleotide searches, the final output is generated by the `lca` module:

The taxonomy format produced by the `lca` module consists of a single taxonomy numeric identifier, followed by a taxonomic rank column and taxonomic name column. The format can contain extra columns, if for example, the lineage info is requested (`--tax-lineage 1` or `--tax-lineage 2`). Here is an example taxonomy classification of two sequences:

```
1758121 subspecies      Limosa lapponica baueri
\00       no rank unclassified
```

This format can be easily converted to TSV:

```
mmseqs createtsv queryDB taxonomyResult taxonomyResult.tsv
```

Each line of the result file `taxonomyResult.tsv` will contain a tab separated list of 1) query accession, 2) LCA NCBI taxon ID, 3) LCA rank name, and 4) LCA scientific name. Any requested additional taxonomy result columns will be included following these four fields.

The `--lca-ranks` parameter can be supplied with a comma (`,`) separated string of taxonomic ranks. For example, `--lca-ranks genus,family,order,superkingdom` will resolve the respective ranks of the LCA and return a semicolon concatenated string of taxa as the fifth column of the result file. Here is an example output.

```
NB501858:55:HMHW7BGXB:1:23301:17888:3880        8932    species Columba livia
NB501858:55:HMHW7BGXB:3:12402:9002:13498        131567  no rank cellular organisms
NB501858:55:HMHW7BGXB:4:23405:2354:17246        299123  subspecies      Lonchura
↪ striata domestica
NB501858:55:HMHW7BGXB:4:11506:25310:7474        117571  no rank Euteleostomi
NB501858:55:HMHW7BGXB:1:21310:9510:6655 0       no rank unclassified
NB501858:55:HMHW7BGXB:1:11112:6821:9848 1758121 subspecies      Limosa lapponica
↪ baueri
NB501858:55:HMHW7BGXB:2:22303:18627:2744        2182385 species Brachybacterium
↪ endophyticum
NB501858:55:HMHW7BGXB:4:22410:13879:7449        8825    superorder      Neognathae
NB501858:55:HMHW7BGXB:3:13402:20359:7200        97097   species Phaethon lepturus
```

For nucleotide-protein searches (contig taxonomy assignment) the final output is generated by the aggregatetax module, and therefore more context is provided:

The taxonomy format produced by the aggregatetax module consists of (1) a single taxonomy numeric identifier, (2) a taxonomic rank column, (3) taxonomic name column, columns for the number of fragments: (3) retained, (4) taxonomically assigned, and (5) in agreement with the contig label (i.e. same taxid or have it as an ancestor), (5) the support received. The format can contain extra columns, if for example, the lineage info is requested (--tax-lineage 1 or --tax-lineage 2).

The final conversion to TSV, including prepending the respective query accession, can again be done with the createtsv module. Next, you can see an example of this output:

```
NC_001133.9      4932    species Saccharomyces cerevisiae        32      32      30
 ↪  0.890    131567;2759;33154;4751;451864;4890;716545;147537;4891;4892;4893;4930;4932
```

The contig has been assigned at the species level to taxid 4932 (Saccharomyces cerevisiae). The assignment is based on 32 protein fragments, which passed the fast prefilter selection. Of these, all 32 fragments received a label and of these, 30 agree with the label assigned to the contig (i.e., they were assigned to species 4932 or to a strain below it). The fraction of -log(E-value) support of the label is 89%.

Taxonomic ranks

The following taxonomic ranks can be used: > forma, varietas, subspecies, species, species subgroup, species group, subgenus, genus, subtribe, tribe, subfamily, family, superfamily, parvorder, infraorder, suborder, order, superorder, infraclass, subclass, class, superclass, subphylum, phylum, superphylum, subkingdom, kingdom, superkingdom

Taxonomy report in Kraken or Krona style

The taxonomy result can also be summarized in a Kraken-style report using the taxonomyreport module.

```
mmseqs taxonomyreport seqTaxDB taxonomyResult taxonomyResult_report
```

The report shows a taxon tree with read counts and mapped fractions.

```
5.6829  362     362       no rank       0  unclassified
94.3171 6008    43        no rank       1  root
87.8493 5596    126       no rank  131567    cellular organisms
42.5903 2713    79   superkingdom    2759      Eukaryota
32.8257 2091    38        no rank   33154        Opisthokonta
```

```
24.0502 1532    2         kingdom   33208         Metazoa
23.8776 1521    3         no rank    6072           Eumetazoa
23.2810 1483    49        no rank   33213             Bilateria
14.2857 910     2         no rank   33511               Deuterostomia
13.9560 889     3          phylum    7711                 Chordata
13.3124 848     0       subphylum   89593                   Craniata
```

The column are (1) the percent of reads covered by the clade rooted at this taxon, (2) number of reads covered by the clade rooted at this taxon, (3) number of reads assigned directly to this taxon, (4) rank, (5) taxonomy identifier, and (6) scientific name. See kraken documentation.

This report can be visualized using the interactive metagenomics data explorer Pavian:

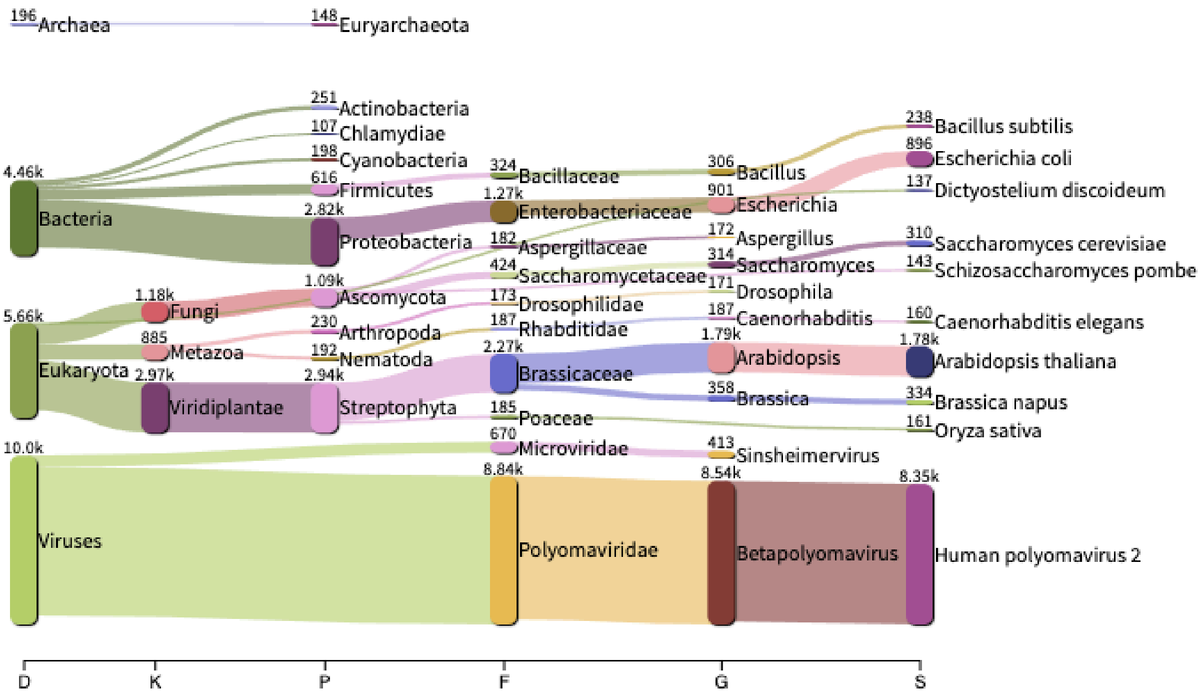

Figure 0.9: Pavian Screenshot

With `--report-mode 1` an interactive Krona based taxonomy report can be created:

```
mmseqs taxonomyreport seqTaxDB taxonomyResult report.html --report-mode 1
```

The resulting `report.html` file can be opened in any modern web browser. It will look similar to the following screenshot:

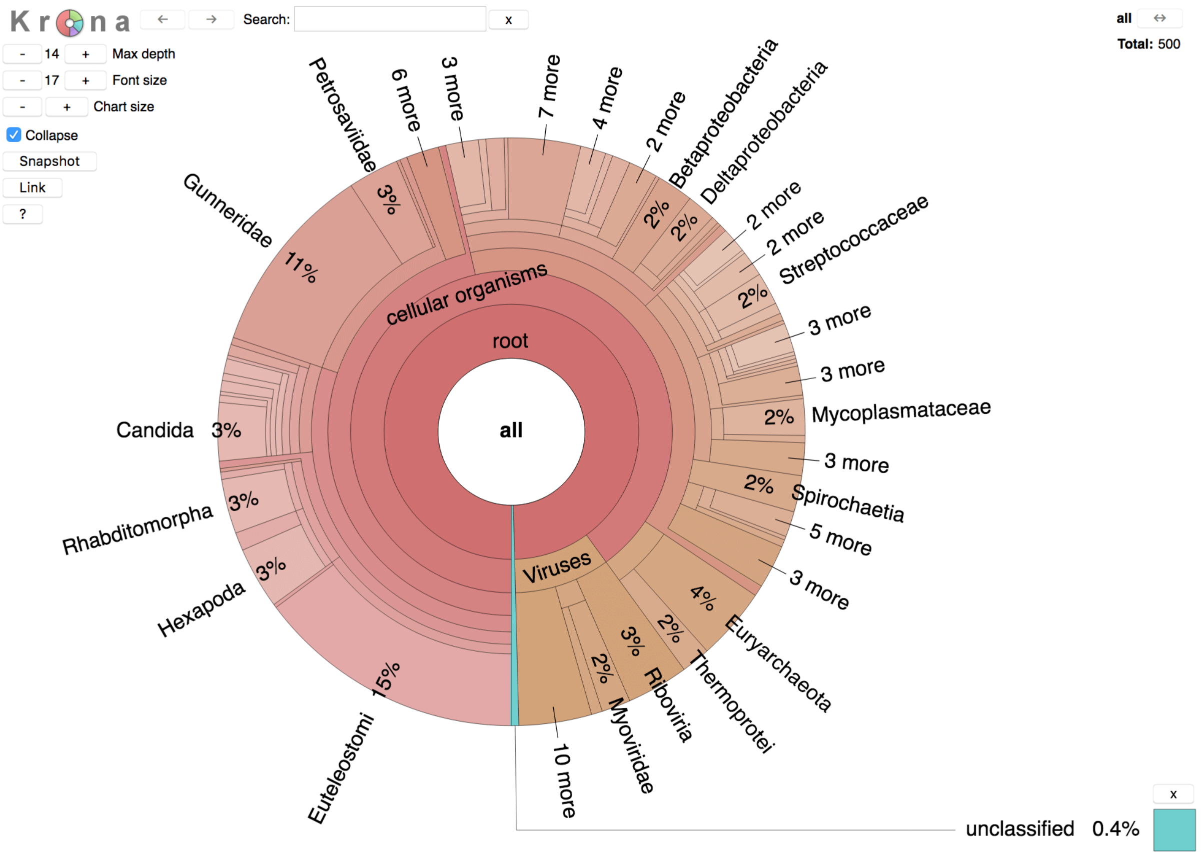

Figure 0.10: Screenshot of a Krona based taxonomy report

Taxonomy top hit report

`easy-taxonomy` produces a top hit report (suffix `tophit_report`), which summarizes the coverages over targets. This report should help to identify spurious alignments. Alignments with high average coverage but low unique coverage have a high chance to be spurious.

The columns are the following:

```
(1) Target identifier
(2) Number of sequences aligning to target
(3) Unique coverage of target uniqueAlignedResidues / targetLength
(4) Target coverage alignedResidues / targetLength
(5) Average sequence identity
(6) Taxonomical information identifier, species, lineage
```

Here is an example output:

```
A0A6B9SVR4 6 0.744 1.026 0.419 112596 species Wolbachia phage WO
↪ d_Viruses;-_Duplodnaviria;-_Heunggongvirae;p_Uroviricota;c_Caudoviricetes;o_Caudovirales;f_Myovi
↪ Myoviridae;s_Wolbachia phage WO
```

Six independent queries align to A0A6B9SVR4. A total of 74.4% of its residues are covered by at least one residue, while the average coverage is 1.026. The average sequence identity is 41.9%.

Filtering taxonomy output

Other MMseqs2 modules work with taxonomyResult databases e.g. `filtertaxdb` can be used to extract taxa, `addtaxonomy` to augment a result database with taxonomic information.

Here is an example for using `filtertaxdb` to extract only results whose taxonomic assignment is any kind of virus (i.e., in the superkingdom "viruses").

```
mmseqs filtertaxdb seqTaxDB taxonomyResult taxonomyResult.virus --taxon-list 10239
awk '$3 != 1 {print}' taxonomyResult.virus > taxonomyResult.virus.id
mmseqs createsubdb taxonomyResult.virus.id queryDB queryDB.virus
mmseqs createsubdb taxonomyResult.virus.id queryDB_h queryDB.virus_h
```

Taxonomy annotation of search/cluster results

Taxonomic annotations of target sequences can be easily added to result files in multiple ways:

- `convertalis` supports multiple taxonomy related output fields (`--format-output taxid,taxname,taxlineage`).
- `addtaxonomy` appends taxonomy annotation columns to an existing taxonomy result database. This is useful for downstream processing (e.g. after conversion to TSV with `createtsv` or `prefixid --tsv` and with external software with `apply`.
- `taxonomyreport` also supports creating reports based on all target database hits found from search results (or cluster members, if given a clustering of seqTaxDB input) or on all sequences in a sequence database.

Create a seqTaxDB from an existing BLAST database

It is easy to create a seqTaxDB from a pre-existing local BLAST databases, if BLAST+ is installed. The following example creates an MMSeqs2 database from NCBI's nt database, but it also works with any of the other BLAST databases including the nr protein database.

First, manually download the NCBI taxonomy database dump:

```
wget ftp://ftp.ncbi.nlm.nih.gov/pub/taxonomy/taxdump.tar.gz
mkdir taxonomy && tar -xxvf taxdump.tar.gz -C taxonomy
```

BLAST+'s blastdbcmd can be used to extract both the FASTA as well as the taxonomy mapping files:

```
blastdbcmd -db nt -entry all > nt.fna
blastdbcmd -db nt -entry all -outfmt "%a %T" > nt.fna.taxidmapping
```

Finally, the createdb and createtaxdb modules use the information to create a complete MMSeqs2 database:

```
mmseqs createdb nt.fna nt.fnaDB
mmseqs createtaxdb nt.fnaDB tmp --ncbi-tax-dump taxonomy/ --tax-mapping-file
 ↪  nt.fna.taxidmapping
```

Create a seqTaxDB for SILVA

If you want to classify ribosomal RNA (16S, 18S, SSU) sequences against the SILVA database you can download it through the databases workflow:

```
mmseqs databases SILVA silvadb tmp
```

To understand how a SILVA MMseqs2 taxonomy sequence database is built take a look at the example script below. We first need to createa NCBI like taxonomy from the SILVA taxonomy.

```
# build name.dmp, node.dmp from SILVA taxonomy
mkdir taxonomy/ && cd "$_"
wget ftp://ftp.arb-silva.de/current/Exports/taxonomy/tax_slv_ssu_*.txt.gz
buildNCBITax=$(cat << 'EOF'
BEGIN{
  ids["root"]=1;
  print "1\t|\t1\t|\tno rank\t|\t-\t|" > "nodes.dmp"
  print "1\t|\troot\t|\t-\t|\tscientific name\t|" > "names.dmp";
}
{ n=split($1, a, ";");
  gsub("domain", "superkingdom", $3);
  ids[$1]=$2;
  gsub(/[^,;]*;$/,"",$1);
  pname=$1;
  if(n==2){
    pname="root"
  }
  pid=ids[pname];
  printf("%s\t|\t%s\t|\t%s\t|\t-\t|\n", $2, pid, $3) > "nodes.dmp";
  printf("%s\t|\t%s\t|\t-\t|\tscientific name\t|\n",$2,a[n-1]) >"names.dmp";
}
```

```
EOF
)
awk -F'\t' "$buildNCBITax" <(gunzip -c tax_slv_ssu_*.txt.gz)
touch merged.dmp
touch delnodes.dmp
cd ..

# create the database SILVA database from Nr99 fasta
wget
↪  ftp://ftp.arb-silva.de/current/Exports/SILVA_*_SSURef_NR99_tax_silva_full_align_trunc.fasta.gz
mmseqs createdb SILVA_*_SSURef_NR99_tax_silva_full_align_trunc.fasta.gz SILVA_DB

# add taxonomy to SILVA_DB
wget ftp://ftp.arb-silva.de/current/Exports/taxonomy/tax_slv_ssu_*.acc_taxid
mmseqs createtaxdb SILVA_DB tmp --ncbi-tax-dump taxonomy/ --tax-mapping-file
↪  tax_slv_ssu_*.acc_taxid
```

Create a seqTaxDB for GTDB

The Genome Taxonomy Database (GTDB) is a phylogenticly consistent database, which redefines the taxonomic tree. MMseqs2 can search against the GTDB but it requires some preprocessing steps.

```
# build name.dmp, node.dmp from GTDB taxonomy
mkdir taxonomy/ && cd "$_"
wget https://data.ace.uq.edu.au/public/gtdb/data/releases/latest/ssu.fna
buildNCBITax=$(cat << 'EOF'
BEGIN{
  ids["root"]=1;
  rank["c"]="class"l
  rank["d"]="superkingdom";
  rank["f"]="family";
  rank["g"]="genus";
  rank["o"]="order";
  rank["p"]="phylum";
  rank["s"]="species";
  taxCnt=1;
  print "1\t|\t1\t|\tno rank\t|\t-\t|" > "nodes.dmp"
  print "1\t|\troot\t|\t-\t|\tscientific name\t|" > "names.dmp";
}
/^>/{
  str=$2
  for(i=3; i<=NF; i++){ str=str" "$i}
```

```
  n=split(str, a, ";");
  prevTaxon=1;
  for(i = 1; i<=n; i++){
    if(a[i] in ids){
      prevTaxon=ids[a[i]];
    }else{
      taxCnt++;
      split(a[i],b,"_");
      printf("%s\t|\t%s\t|\t%s\t|\t-\t|\n", taxCnt, prevTaxon, rank[b[1]]) >
       ↪  "nodes.dmp";
      printf("%s\t|\t%s\t|\t-\t|\tscientific name\t|\n", taxCnt, b[3]) >"names.dmp";
      ids[a[i]]=taxCnt;
      prevTaxon=ids[a[i]];
    }
  }
  gsub(">", "", $1);
  printf("%s\t%s\n", $1, ids[a[n]]) > "mapping";
}
EOF
)
awk -F'\\[loc' '{ print $1}' ssu.fna | awk "$buildNCBITax"
touch merged.dmp
touch delnodes.dmp
cd ..

mmseqs createdb ssu.fna ssu

# add taxonomy to GTDB
mmseqs createtaxdb ssu tmp --ncbi-tax-dump taxonomy/ --tax-mapping-file
 ↪  taxonomy/mapping
```

When searching against the GTDB disable the default set blacklisted clades by specifying `--blacklist ""` parameters. Here the clades of all NCBI unclassified sequences and other sequences (plasmids, etc.) are ignored in LCA computation. As GTDB uses taxonomic IDs that are not compatible to the NCBI's, the default blacklist cannot be used.

Create a seqTaxDB by manual annotation of a sequence database

Here is an example how to manually annotate a sequence database with taxonomic information. The example uses Uniprot identifiers.

As a first step turn the FAST[A/Q] file into a mmseqs sequence database using `createdb`

```
# Turn the sequences into a MMseqs2 database (this also creates sequenceDB.lookup)
```

```
# Skip this step if you already created a database
mmseqs createdb sequence.fasta sequenceDB
```

`createdb` produces a tab-separated sequenceDB.lookup file that contains numeric-db-id, Accession (e.g. Uniprot Accession Q6GZX4) and File. IDs are parsed from the header from the input database (see id parsing from headers).

```
0 Q6GZX4  0
1 Q6GZX3  0
2 Q197F8  0
3 P0A031  0
4 Q197F7  0
```

As next step, we create a tab-separated mapping with every target database identifier mapped to a NCBI taxon identifier. The mapping file should be in the format `Accession numeric-ncbi-tax-id`.

```
Q6GZX4    654924
Q6GZX3    654924
Q197F8    345201
Q197F7    345201
```

Here is an example how to transform an Uniprot mapping file into the tab-separated mapping file.

```
# The taxidmapping file should be in the format
# Accession numeric-ncbi-tax-id
# Q6GZX4    654924
# Q6GZX3    654924
# Q197F8    345201
# Q197F7    345201

# e.g. download the uniprot mapping file and convert it to the taxidmapping mapping
↪  format
URL="ftp://ftp.uniprot.org/pub/databases/uniprot/current_release/knowledgebase/idmapping/idmapping.da
wget -nv -O - "$URL" | zcat | awk '$2 == "NCBI_TaxID" {print $1"\t"$3 }' >
↪  taxidmapping
```

We need the NCBI taxonomy taxdump.tar.gz. It is available on the NCBI FTP server:

```
mkdir ncbi-taxdump && cd ncbi-taxdump
wget ftp://ftp.ncbi.nih.gov/pub/taxonomy/taxdump.tar.gz
tar xzvf taxdump.tar.gz
cd -
```

As a final step we can now use `createtaxdb` to annotate our sequence database.

```
# now we can use createtaxdb with our own mapping.
mmseqs createtaxdb sequenceDB tmp --ncbi-tax-dump ncbi-taxdump --tax-mapping-file
 ↪  taxidmapping
```

It is possible to inspect how many identifiers have an assigned taxon with the following code

```
awk 'FNR==NR{f[$1]=$2; next} $1 in f{ print $2" has taxid "f[$1];} !($1 in f){print
 ↪  $2" has no taxid";} ' sequenceDB_mapping sequenceDB.lookup
```

Reciprocal best hit using `mmseqs rbh`

Given two protein sets 'A' and 'B', MMseqs2 can search in two directions and report pairs ('a','b'), such that 'b' is the highest scoring hit of 'a' and 'a' is the highest scoring hit of 'b':

```
mmseqs easy-rbh Aproteins.fasta Bproteins.fasta ABrbh tmp
```

Behind the scenes

The workflow searches with each 'a' against the targets in 'B'. It retains for each 'a' the maximal bitscore it got with any of its hits (denoted 'x'). It then searches with each 'b' against targets in A and retains its best scoring hit(s). If among these, 'a' is found with the score 'x', the pair ('a','b') will be reported as an RBH pair. Importantly, no correction for composition bias nor low complexity masking is performed during the searches (`--comp-bias-corr 0` and `--mask 0`) to assure the bitscores remain the same irrespective of the search direction. Finally, please note that E-values are not used to determine pairing but they are used as a cutoff for the searches (`rbh` uses the default value of `-e` and this can be changed, if needed).

Description of core modules

For advanced users, it is possible to skip the workflows and execute the core modules for maximum flexibility. Especially for the sequence search it can be useful to adjust the prefiltering and alignment parameters according to the needs of the user.

MMseqs2 contains three core modules: prefiltering, alignment and clustering.

Computation of prefiltering scores using `mmseqs prefilter`

The prefiltering module computes an ungapped alignment score for all consecutive k-mer matches between all query sequences and all database sequences and returns the highest score per sequence.

If you want to *cluster* a database, or do an all-against-all search, the same database will be used on both the query and target side. the following program call does an all-against-all prefiltering:

```
mmseqs prefilter sequenceDB sequenceDB resultDB_pref
```

`sequenceDB` is the base name of the MMseqs2 databases produced from the FASTA sequence databases by `mmseqs createdb`, the prefiltering results are stored in the MMseqs2 database files `resultDB_pref` and`prefilterDB.index`.

For *sequence search* two different input databases are usually used: a query database `queryDB` and a target database `targetDB`, though they can again be identical. In this case, the prefiltering program call is:

```
mmseqs prefilter queryDB targetDB resultDB_pref
```

MMseqs2 can handle profiles or protein/nucleotide sequences as input for the `queryDB`.

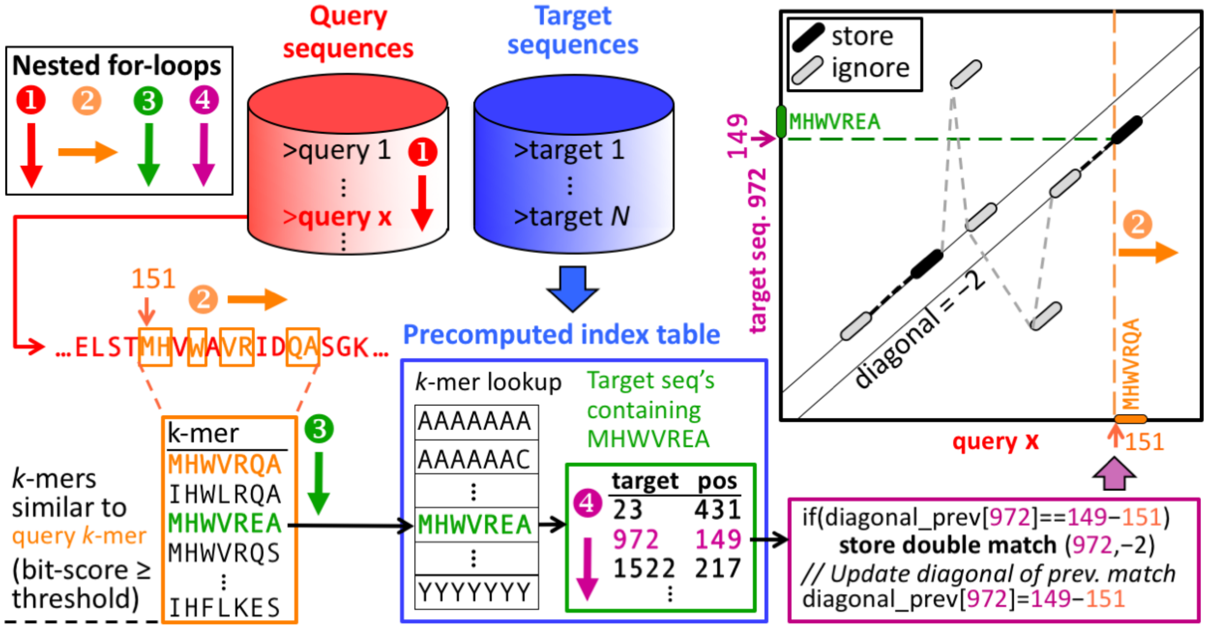

Figure 0.11: Prefilter

The prefilter k-mer match stage is key to the high speed and sensitivity. It detects consecutive short words ("k-mer") match on the same diagonal. The diagonal of a k-mer match is the difference between the positions of the two similar ''k' '-mer in the query and in the target sequence.

The pre-computed index table for the target database (blue frame) contains for each possible ''k' '-mer the list of the target sequences and positions where the k-mer occurs (green frame).

Query sequences/profiles are processed one by one (loop 1). For each overlapping, spaced query k-mer (loop 2), a list of all similar k-mer is generated (orange frame). The similarity threshold

determines the list length and sets the trade-off between speed and sensitivity. The similar k-mer list length can be controlled with `-s`.

For each similar k-mer (loop 3) we look up the list of sequences and positions where it occurs (green frame). In loop 4 we detect consecutive double matches on the same diagonals (magenta and black frames).

For each consecutive k-mer matches an ungapped alignment is computed. Only the maximal ungapped alignment score for each target is reported.

Set sensitivity `-s` parameter

The sensitivity of the prefiltering can be set using the `-s` option. Internally, `-s` sets the average length of the lists of similar k-mers per query sequence position.

- *Similar k-mers list length*: Low sensitivity yields short similar k-mer lists. Therefore, the speed of the prefiltering increases, since only short k-mer lists have to be generated and less lookups in the index table are necessary. However, the sensitivity of the search decreases, since only very similar k-mers are generated and therefore, the prefiltering cannot identify sequence pairs with low sequence identity.

It is possible to speed best hits searches by stepwise increasing `-s`. MMseqs2 includes a workflow for this purpose. How to find the best hit the fastest way

The following graphic shows the average AUC sensitivity versus speed-up factor relative to BLAST for 637,000 test searches. White numbers in plot symbols give number of search iterations.

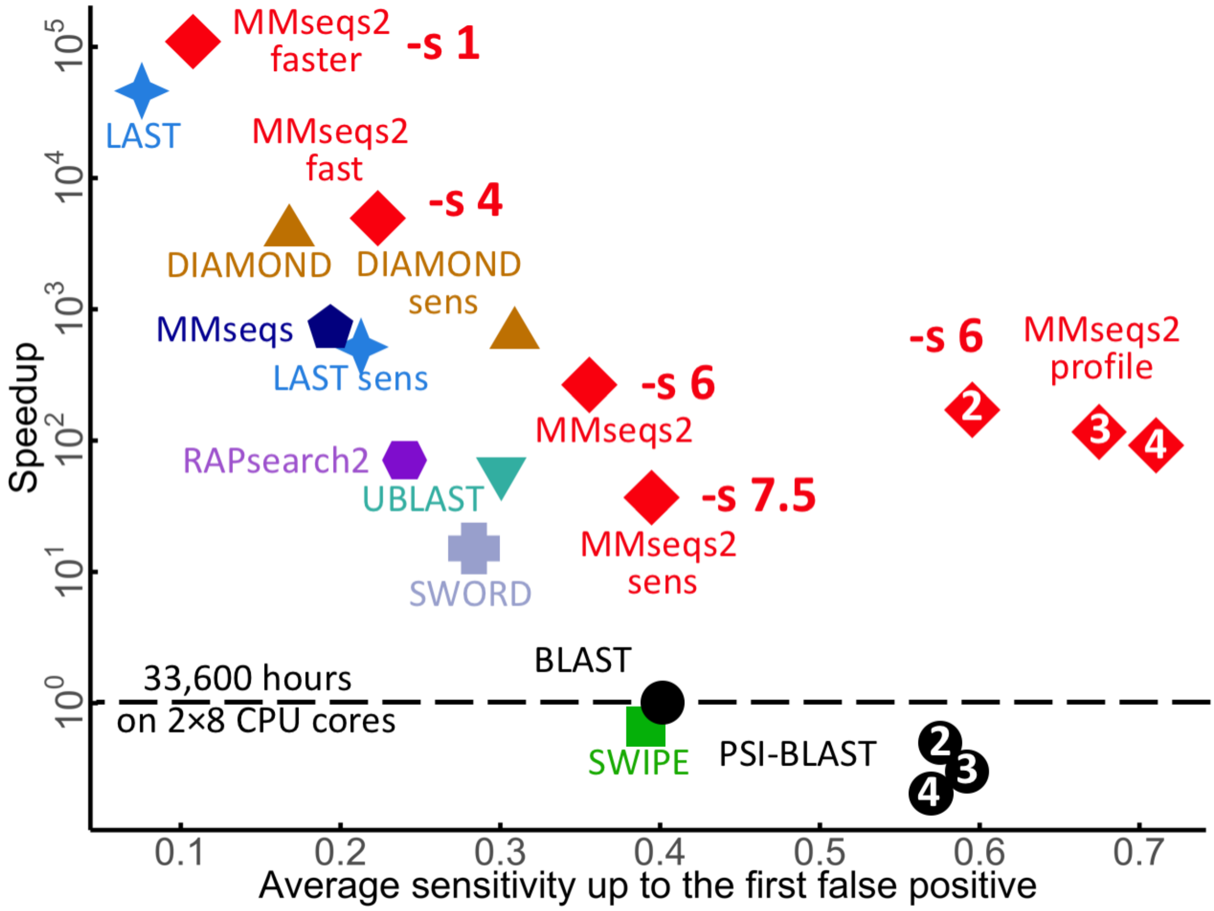

Figure 0.12: Prefilter sensitivity vs. speed

It is furthermore possible to use change the k-mer lengths, which are used in the prefiltering. Longer k-mers are more sensitive, since they cause less chance matches. Though longer k-mers only pay off for larger databases, since more time is needed for the k-mer list generation, but less time for database matching. Therefore, the database matching should take most of the computation time, which is only the case for large databases. As default MMseqs2 ties to compute the optimal k-mer length based on the target database size.

Local alignment of prefiltered sequence pairs using `mmseqs align`

In the alignment module, you can also specify either identical or different query and target databases. If you want to do a clustering in the next step, the query and target databases need to be identical:

```
mmseqs align sequenceDB sequenceDB resultDB_pref resultDB_aln
```

Alignment results are stored in the database files `resultDB_aln` and `resultDB_aln.index`.

Program call in case you want to do a sequence search and have different query and target databases:

```
mmseqs align queryDB targetDB resultDB_pref resultDB_aln
```

This module implements a SIMD accelerated Smith-Waterman-alignment (Farrar, 2007) of all sequences that pass a cut-off for the prefiltering score in the first module. It processes each sequence pair from the prefiltering results and aligns them in parallel, calculating one alignment per core at a single point of time. Additionally, the alignment calculation is vectorized using SIMD (single instruction multiple data) instructions. Eventually, the alignment module calculates alignment statistics such as sequence identity, alignment coverage and e-value of the alignment.

Clustering sequence database using `mmseqs clust`

For calling the stand-alone clustering, you need the input sequence database and a result database:

```
mmseqs cluster sequenceDB resultsDB_aln resultsDB_clu
```

Clustering results are stored in the MMseqs database files `resultsDB_clu` and `resultsDB_clu.index`.

The clustering module offers the possibility to run three different clustering algorithms by altering the `--cluster-mode` parameter. A greedy set cover algorithm is the default (`--cluster-mode 0`). It tries to cover the database by as few clusters as possible. At each step, it forms a cluster containing the representative sequence with the most alignments above the special or default thresholds with other sequences of the database and these matched sequences. Then, the sequences contained in the cluster are removed and the next representative sequence is chosen.

The second clustering algorithm is a greedy clustering algorithm (`--cluster-mode 2`), as used in CD-HIT. It sorts sequences by length and in each step forms a cluster containing the longest sequence and sequences that it matches. Then, these sequences are removed and the next cluster is chosen from the remaining sequences.

The third clustering algorithm is the connected component algorithm. This algorithm uses the transitivity of the relations to form larger clusters with more remote homologies. This algorithm adds all sequences to a cluster, that are reachable in a breadth first search starting at the representative with the most connections.

File formats

MMseqs2 database format

Most MMseqs2 modules consume and produce files in the MMseqs2 database format. The format is inspired by ffindex (https://github.com/soedinglab/ffindex_soedinglab), which was developed by

Andreas Hauser. It avoids drastically slowing down the file system when millions of files would need to be written or accessed, e.g. one file per query sequence in a many-to-many sequence search. MMseqs2 databases hide these files from the file system by storing them in a single data file. The *data file* `<name>` contains the data records, i.e. the contents of the file, concatenated and separated by `\0` characters. A second, *index file* `<name>.index` contains for each numerical identifies (corresponding to the file name) the position of the corresponding data record in the data file. The `<name>.dbtype` contains the database type e.g. Protein, Nucleotide, ...

Each line of the *index file* contains, separated by tabs, (1) the ID, (2) the offset in bytes of the data_record counted from the start of the data file, and (3) the size of the data record. The IDs have to be sorted numerically in ascending order, since for accessing a data record by IDs the matching IDs are found by binary search.

Here is an example for a database containing four sequences:

```
PSSLDIRL
\0GTLKRLSAHYTPAW
\0AEAIFIHEG
\0YTHGAGFDNDI
\0
```

The corresponding index file (file extension `.index`) could look like this.

```
10  0   9
11  9  15
12  24  10
13  34  12
```

The index contains four IDs, one for each data record: 10, 11, 12 and 13. The corresponding data records have offset positions 0, 9, 25, 35 and the data record sizes are 9, 15, 10, and 12 respectively.

Databases are accompanied by dbtype file (extension `.dbtype`). For sequence databases there are three db types: amino acid, nucleotide and profile. The dbtype just contains a number in binary format. In case the `.dbtype` is missing it is possible to create a `.dbtype` file with the following commands.

```
# Amino acid sequence database
awk 'BEGIN { printf("%c%c%c%c",0,0,0,0); exit; }' > seqDb.dbtype
# Nucleotide sequence database
awk 'BEGIN { printf("%c%c%c%c",1,0,0,0); exit; }' > seqDb.dbtype
# Profile database
awk 'BEGIN { printf("%c%c%c%c",2,0,0,0); exit; }' > seqDb.dbtype
# Generic database e.g for header databases (extension '_h')
awk 'BEGIN { printf("%c%c%c%c",12,0,0,0); exit; }' > seqDb.dbtype
```

The MMseqs2 modules `createdb` converts from FASTA/FASTQ[.gz|.bz] to the MMseqs2 database format. `createdb` generates an MMseqs2 database from a FASTA sequence database. It assigns each sequence in the file a numerical identifier and shuffles the database. MMseqs2 sequence database can be converted back to a fasta database by `convert2fasta`.

However, for fast access in very large databases it is advisable to use the MMseqs2 database directly without converting it to FASTA format.

Manipulating databases

The data file of the databases cannot be altered easily since any change would break the offset in the `.index` file. MMseqs2 module create new databases rather than changing existing ones. We have a heap of modules to manipulate MMseqs2 database, such as `createsubdb`, `filterdb`, `concatdbs`, `mergedbs` and `apply`.

Altering the content of the `.index` file is possible. You can, for example, create a subset of the index. This mechanism could be used to create a database with only sequence longer than 100 residues.

```
mmseqs createdb seqDb.fas seqDb
# here we select member that are greater 100 (also count the newline and null bytes)
awk '$3 > 102 {print $1}' seqDb.index > ids.gt100
mmseqs createsubdb ids.gt100 seqDb seqDb.gt100
mmseqs createsubdb ids.gt100 seqDb_h seqDb.gt100_h
```

Sequence database format

The sequence database consists of two databases the sequence data and the header. `createdb` takes an FASTA/FASTQ[.gz|.bz] as input and creates two six files. Each line in the sequence data file is a separate sequence followed by an null byte `\0`. E.g. the following data file has four sequences.

```
MPNGASLCVRFRAHGDAPFFSRD
\0MEVVERHAMFGGEMLTFTHLSASCGFAM
\0MSSQTVSEVVNEYVGPADGVCVAVVGAT
\0MAVALELISQH
\0
```

The corresponding index file (file extension `.index`) looks like this. The first column is the numeric sequence identifier, second the offset and third the length. The length contains the null byte and the new line. The real sequence length is two characters shorter (`$3 - 2`).

```
0	0	25
1	25	30
```

```
2       55      30
3       85      13
```

The header database (file ending `_h`) contains the data of > entries in FASTA and `@` entries in FASTQ files.

```
tr|Q0KJ32|Q0KJ32_9ACTO Aspartate semialdehyde dehydrogenase OS=Streptomyces albulus
 ↪  GN=asd PE=3 SV=1
\0tr|F0YHT8|F0YHT8_9STRA Putative uncharacterized protein OS=Aureococcus
 ↪  anophagefferens GN=AURANDRAFT_31056 PE=4 SV=1
\0tr|C0XU54|C0XU54_9CORY Aspartate-semialdehyde dehydrogenase OS=Corynebacterium
 ↪  lipophiloflavum DSM 44291 GN=asd PE=3 SV=1
\0tr|D6KVP9|D6KVP9_SCAIO Aspartate-semialdehyde dehydrogenase OS=Scardovia inopinata
 ↪  F0304 GN=HMPREF9020_01065 PE=3 SV=1
```

The header index has also four entries. The identifier in the first column correspond to identifier in the index of the data file.

```
0       0       102
1       102     118
2       220     123
3       343     118
```

Sequence database can be converted back to FASTA only with `convert2fasta`

```
mmseqs convert2fasta seqDb seqDb.fasta
```

Prefiltering format

Each data record consists of the prefilter results for one query sequence. The ID is the database accession code, a numerical identifier (ID) for the query that was assigned by `createdb`.

Each line in a data record reports on one matched database sequence and has the following format (white space between columns contains one tab character):

```
targetID  ungappedScore  diagonal
```

where `targetID` is the database identifier of the matched sequence, the ungapped score of the match, and `diagonal` is the diagonal i-j (i = position in query, j = position in db sequence) on which the match occurs.

Example of a database record for prefiltering:

```
0       71      0
2       35      0
3       15      8
```

The first line describes a match with database sequence 2 on `diagonal` 0 with a `-log(e-value)` of 71 (e-value 1.46e-31).

Prefilter database can be converted to TSV by `createtsv`.

```
mmseqs createtsv queryDB targetDB prefRes prefRes.tsv
```

Each line of The TSV represents a single entry in the prefilter result. The first column contains then the query, target identifier, ungapped score and diagonal respectively.

```
Q0KJ32  Q0KJ32  71   0
Q0KJ32  C0W539  35   0
Q0KJ32  D6KVP9  15   8
```

Alignment format

Most of the time user will use the alignment format provided by `convertalis` (see Custom alignment format with convertalis). Main difference between internal and external format is, the internal format is 0-indexed and the external is 1-indexed. To understand the internal alignment produced by the module `align`, `alignbykmer` or `rescorediagonal --rescore-mode 2` read the next section.

Internal alignment format

Each data record consists of the alignment results for one query sequence. The ID of the queries was assigned by `createdb`.

Each line in a data record reports on match, i.e., one database sequence aligned to the query. It has the following format (white space between columns contains one tab character):

```
targetID  alnScore  seqIdentity  eVal  qStart  qEnd  qLen  tStart  tEnd  tLen
 ↪  [queryOrfStart] [queryOrfEnd] [dbOrfStart] [dbOrfEnd] [alnCigar]
```

Here, `targetID` is the database identifier of the matched sequence, `alnScore` is the bit score of the alignment in half bits, `seqIdentity` is the sequence identity [0:1], `eVal` is the e-value of the match, `qStart` is the start position of the alignment (0-indexed) in the query, `qEnd` is the end position of the alignment (0-indexed) in the query, `qLen` is the length of the query, `tStart` and `tEnd` are the start and end positions (0-indexed) in the target (i.e. the database sequence), `tLen` is the target sequence length, `queryOrfStart`,`queryOrfEnd`,`dbOrfStart` and `dbOrfEnd` are the start and end position of the orf extracted by `extractorfs`. This fields added by `offsetalignment` the optional `alnCigar` string encodes the alignment in compressed format and is only included in the results if the option `-a` was used in MMseqs2 search. The numbers preceding the three letters M, I, and D give the number of match positions in a block aligned without gaps, the number of insertions and of deletions, respectively.

Example data record for alignment results:

```
0    734 1.000   2.084E-243  0    378 379 0    378 379 379M
2    260 0.455   1.305E-79   26   368 379 21   363 369 173M2D41M2D65M6I21M2D37M
3    233 0.434   2.830E-70   25   364 379 30   367 373
 ↪  162M2I16M3I10M1I5M6D16M2D67M6I25M2D27M
```

The first line with targetID 2 is an identity match. The last sequence 3 has a Smith-Waterman alignment score of 347, the sequence identity 0.565 and the e-value 2.722e-99, the query start and end position is 13,367 of the total length 373, the target start and end position is 20,367 of the total length 373, the alignment string is 10M5I53M3I118M1D166M.

The alignment result can be converted into a flat file by `createtsv` or `convertalis`.

```
Q0KJ32  Q0KJ32  783 1.000   7.540E-260  0    418 419 0    418 419 419M
Q0KJ32  C0W539  260 0.455   1.305E-79   26   368 379 21   363 369
 ↪  173M2D41M2D65M6I21M2D37M
Q0KJ32  D6KVP9  233 0.434   2.830E-70   25   364 379 30   367 373
 ↪  162M2I16M3I10M1I5M6D16M2D67M6I25M2D27M
```

Custom alignment format with convertalis

An alignment result database can be converted into human readable format with the `convertalis` module.

```
mmseqs convertalis queryDB targetDB alnRes alnRes.tab
```

By default (`--format-mode 0`), `alnRes.tab` will contain alignment result in a BLAST tabular result (comparable to `-m 8 -outfmt 6`) with 12 columns: (1,2) identifiers for query and target sequences/profiles, (3) sequence identity, (4) alignment length, (5) number of mismatches, (6) number of gap openings, (7-8, 9-10) domain start and end-position in query and in target, (11) E-value, and (12) bit score.

The option `--format-output` defines a custom output format. For example, the format string `--format-output "query,target,evalue,qaln,taln"` prints the query and target identifiers, e-value of the alignment and the alignments.

Column headers can be added to the output with `--format-mode 4`. This mode also supports chosing a custom output format.

The following field are supported

- **query** Query sequence identifier
- **target** Target sequence identifier
- **evalue** E-value

- **gapopen** Number of gap open events (note: this is NOT the number of gap characters)
- **pident** Percentage of identical matches
- **fident** Fraction of identical matches
- **nident** Number of identical matches
- **qstart** 1-indexed alignment start position in query sequence
- **qend** 1-indexed alignment end position in query sequence
- **qlen** Query sequence length
- **tstart** 1-indexed alignment start position in target sequence
- **tend** 1-indexed alignment end position in target sequence
- **tlen** Target sequence length
- **alnlen** Alignment length (number of aligned columns)
- **raw** Raw alignment score
- **bits** Bit score
- **cigar** Alignment as string. Each position contains either M (match), D (deletion, gap in query), or I (Insertion, gap in target)
- **qseq** Query sequence
- **tseq** Target sequence
- **qaln** Aligned query sequence with gaps
- **taln** Aligned target sequence with gaps
- **qheader** Header of Query sequence
- **theader** Header of Target sequence
- **qframe** Query frame (-3 to +3)
- **tframe** Target frame (-3 to +3)
- **mismatch** Number of mismatches
- **qcov** Fraction of query sequence covered by alignment
- **tcov** Fraction of target sequence covered by alignment
- **empty** Dash column '-'
- **taxid** Taxonomical identifier (needs mmseqs tax db)
- **taxname** Taxon Name (needs mmseqs tax db)
- **taxlineage** Taxonomical lineage (needs mmseqs tax db)
- **qset** Query filename of FASTA/Q (useful if multiple files were passed to createdb)
- **qsetid** Numeric identifier for query filename
- **tset** Target filename of FASTA/Q (useful if multiple files were passed to createdb)
- **tsetid** Numeric identifier for target filename

We support output in SAM format using `--format-mode 1`: the AS tag contains the raw score, NM is the mismatch count.

`--format-mode 3` will return an interactive HTML document to visualize search results. The in-

put alignment result needs to contain backtraces (i.e. the search must have been run with the -a parameter).

Clustering format

Internal cluster format

Each data record consists of the IDs of the members of one cluster. The ID refers to the representative sequence of that cluster, (usually assigned by `createdb`).

Each line in a data record contains one ID of a cluster member. The first line of each data record contains the ID of the representative sequence of that cluster.

Here is an example of two clusters, the first has 3 cluster members (0,2,3) the second 2 cluster member (5,1)

```
0
2
3
\05
1
```

In the first cluster the 0 is the ID of the representative sequence while 2 and 3 are cluster members. In the second cluster 5 is the representative sequence and 1 is a member

Cluster TSV format

The internal format can be converted to a flat tsv file:

```
mmseqs createtsv sequenceDB sequenceDB resultsDB_clu resultsDB_clu.tsv
```

The `resultsDB_clu.tsv` file follows the following format:

```
#cluster-representative     cluster-member
Q0KJ32  Q0KJ32
Q0KJ32  C0W539
Q0KJ32  D6KVP9
E3HQM9  E3HQM9
E3HQM9  F0YHT8
```

All members of the clustering are listed line by line. Each cluster is a consecutive block in the file. The first column always contains the representative sequence, the second contains the cluster member. For the example the cluster with the representative sequence Q0KJ32 contains four members itself and C0W539, D6KVP9, D1Y890. IDs are parsed from the header from the input database (see id parsing from headers).

Cluster FASTA-like format

The internal format can be converted to a fasta-like format:

```
mmseqs createseqfiledb DB clu clu_seq
mmseqs result2flat DB DB clu_seq clu_seq.fasta
```

The resulting FASTA-like format file will look like this:

```
>Q0KJ32
>Q0KJ32
MAGA....R
>C0W539
MVGA....R
>D6KVP9
MVGA....R
>D1Y890
MVGV....R
>E3HQM9
>E3HQM9
MCAT...Q
>Q223C0
MCAR...Q
```

A new cluster is marked by two identical name lines of the representative sequence, where the first line stands for the cluster and the second is the name line of the first cluster sequence. It is followed by the fasta formatted sequences of all its members.

Extract representative sequence

To extract the representative of a clustering use the following commands:

```
mmseqs createsubdb DB_clu DB DB_clu_rep
mmseqs convert2fasta DB_clu_rep DB_clu_rep.fasta
```

The resulting fasta will contain all representative sequences:

```
>Q0KJ32
MAGA....R
>E3HQM9
MCAT...Q
```

Taxonomy format

Internal taxonomy format

See: Taxonomy output and TSV

Taxonomy report in Kraken or Krona style

See: Taxonomy report in Kraken or Krona style

LCA TSV

See: Taxonomy output and TSV

Profile format

The MMseqs2 internal profile format contains 23 values stored per position. The first 20 values are the linear probabilities without pseudo counts in the order ACDEFGHIKLMNPQRSTVWY. We compress the floats using the minifloat implementation with 5 mantissa and 3 exponent bits. Value 21, 22, 23 contains the query residue, consensus residue and the Neff value respectively.

Profiles can be transformed into a Blast like PSSM format with the following command.

```
mmseqs profile2pssm profileDB pssmFile
```

The output format is another database with a human readable representation

```
Pos  Cns  A   C   D   E   F   G   H   I   K   L   M   N   P   Q   R   S   T   V   W
 ↪  Y
0    M    -2  -1  -1  -2  -2  -2  -2  -1  -2  -2  10  -1  -1  -2  -2  -1  -1  -2  -2
 ↪  -1
1    A    8   -1  -1  -2  -2  -1  -2  -1  -2  -2  -2  -1  -1  -2  -2  -2  -1  -2  -1
 ↪  -1
2    A    8   -1  -1  -2  -2  -1  -2  -1  -2  -2  -2  -1  -1  -2  -2  -1  -1  -2  -1
 ↪  -1
3    M    -2  -1  -1  -2  -2  -1  -2  -1  -2  3   8   -1  -1  -2  -2  -1  -1  -2  -1
 ↪  -1
4    E    -2  -1  -1  7   -1  -1  -2  -1  4   -2  -2  -1  -1  -2  -2  -2  -1  -2  -1
 ↪  -1
5    L    -2  -1  -1  -2  -1  -1  -2  -1  4   3   -2  -1  -1  -2  -2  -2  -2  4   -1
 ↪  -1
6    I    -2  -1  -1  -2  -1  -1  -2  6   -2  1   -2  -1  -1  -2  -2  -2  -2  3   -1
 ↪  -1
7    E    -2  -1  -1  6   -1  -1  -2  -1  -2  -2  -2  -1  -1  -2  -2  5   -2  -2  -1
 ↪  -1
```

```
8    R    -2  -1  -1  3   -1  -2  -2  -1  -2  -2  -2  -1  -1  5   5   3   -2  -2  -1
 ↪  -1
9    H    -2  -2  -1  -2  -2  -2  11  -1  -2  -2  -2  -1  -1  -1  -1  -2  -2  -2  -1
 ↪  -1
```

By default, `profile2pssm` generates a flat file with a header for each query containing:

```
Query profile of sequence #database_key
```

`#database_key` corresponds to the database key in the `.index` file of the profile database. `profile2pssm` can also output an indexed database with the `--db-output` parameter. In this case, the header is omitted.

There are three ways to use external PSSM in MMseqs2.

Parameters that affect profile construction

`--diff N`: Before a profile is computed, the sequences of the alignments are filtered and only the N most diverse are kept and used for profile computation. `--qid` and `--qsc` also give minimum thresholds for the filtering.

Convert a result database into a profile

All MMseqs2 result database (like clustering, alignment, prefilter results …) can be transformed into profiles with the `result2profile` module.

```
mmseqs result2profile seqDB seqDB resultDB profileDB
```

Examples how to use `result2profile`. Turn a search result into profiles:

```
mmseqs search queryDb targetDb alnDb tmp -a
mmseqs result2profile queryDb targetDb alnDb queryProfileDb
```

Turning a cluster result into profiles :

```
mmseqs cluster sequenceDb clusterDB tmp
mmseqs createsubdb clusterDB sequenceDb sequenceRepDb
mmseqs createsubdb clusterDB sequenceDb_h sequenceRepDb_h
mmseqs result2profile sequenceRepDb sequenceDb clusterDB sequenceRepProfileDb
```

Convert an external MSA into a profile

MMseqs2 can compute profiles from MSAs with the `msa2profile` module. It is possible to use MSAs in FASTA, A3M and CA3M format. In default the first sequence in the MSA is chosen as the query sequence. Gap columns in the query are discarded. But it is also possible to compute a consensus query sequence from the MSA by

```
mmseqs convertmsa stockholm.msa msaDb
mmseqs msa2profile msaDb profileDB
```

The `msa2profile` decide if a column of the multiple sequence alignment will be considered in the profile or not. There are two different modes. The sensitivity of searches with the sequence profiles can depend critically on which columns are represented.

By default, MMseqs2 uses the setting

`--match-mode 0` (profile column assignment by first sequence in MSA),

which means match states are assigned by the first (master) sequence in the MSA: All columns of the first sequence except gaps '-' will be turned into profile columns. This setting can be used for center star MSAs where the first sequence is the center. It is risky for large MSAs in which the first sequence might be not very representative of the entire MSA.

`--match-mode 1` (profile column assignment by gap fraction)

which turns all columns with at least 50% residues (non-gaps) to profile columns and treats all others as insertions. The threshold ratio can be changed with the option

`--match-ratio 0.5` (change gap fraction threshold for profile column assignment).

If you want to use the Pfam database see How to create a target profile database (from PFAM).

Extract consensus or sequence information from a profile

Profiles generated by `msa2profile`, `result2profile`, `convertprofiledb`, etc. also contain the sequence information for the consensus and representative (= first sequence in the alignment) residues for each profile columns.

The consensus sequence can be extracted into a normal MMseqs2 sequence database with the `profile2consensus` module, while the representative sequence can be extracted with the `profile2repseq` module.

Convert HHsuite HMMs into a profile

It is possible to convert the HH-suite HMM format to MMseqs2 profiles with the `convertprofiledb`. This conversation is only possible if the HMMs do not contain any pseudo counts. The HMMs need to be in a MMseqs2 database format. This can be done using `ffindex_build` (https://github.com/soedinglab/ffindex_soedinglab).

```
hhmake -i 1TIM.a3m -o 1TIM.hmm -pc_hhm_nocontxt_mode 0
hhmake -i 6IGF.a3m -o 6IGF.hmm -pc_hhm_nocontxt_mode 0
ffindex_build hmm hmm.index 1TIM.hmm 6IGF.hmm
mmseqs2 convertprofiledb hmm profileDb
```

Identifier parsing

MMseqs2 parses identifier from the fasta header when transforming a result DB into a flat file by using e.g. `createtsv`, `convertalis`, …). We support following fasta header types:

```
Uniclust,
Swiss-Prot,
Trembl,
GenBank,
NCBI Reference Sequence,
Brookhaven Protein Data Bank,
GenInfo Backbone Id,
Local Sequence identifier,
NBRF PIR,
Protein Research Foundation,
General database identifier,
Patents,
NCBI GI
```

If none of the header supported could be detected than we extract everything from header start (excluding >) until the first whitespace.

Optimizing sensitivity and consumption of resources

This section discusses how to keep the run time, memory and disk space consumption of MMseqs2 at reasonable values, while obtaining results with the highest possible sensitivity. These considerations are relevant if the size of your database exceeds several millions of sequences and are most important if the database size is in the order of tens of millions of sequences.

Prefiltering module

The prefiltering module can use a lot of resources (memory consumption, total runtime and disk space), if the parameters are not set appropriately.

Memory consumption

For maximum efficiency of the prefiltering, the entire database should be held in RAM. The major part of memory is required for the k-mer index table of the database. For a database containing `N` sequences with an average length `L`, the memory consumption of the index lists is `(N * L * 7) byte`. Note that the memory consumption grows linearly with the size of the sequence database. In addition, the index table stores the pointer array and two auxiliary arrays with the memory consumption of

a^k*8 byte, where a is the size of the amino acid alphabet (default a=20, does not include the unknown amino acid X) and k is the k-mer size. The overall memory consumption of the index table is

```
M = (7 * N * L + 8 a^k) byte
```

Therefore, the UniProtKB database version of April 2014 containing 55 million sequences with an average length 350 needs about 71 GB of main memory.

If not enough memory is available to hold the whole database MMseqs2 will automatically split the target database.

The `--split-memory-limit` parameter can give MMseqs2 an upper limit of system RAM to use for the large prefiltering data structures. MMseqs2 will still use some additional memory for its database structures etc. In total, `--split-memory-limit` will be about 80% of the total memory required. Order of magnitude suffices can be passed to `--split-memory-limit`, such as `10G` for ten gigabyte or `1T` for one terabyte of RAM.

Database splitting runtime slowdown

Target database splitting primarily affects MMseqs2's runtime by introducing an additional result merging stage. The slowdown is less noticable on higher sensitivity levels, where the actual prefiltering dominates runtime and most noticable on lower sensitivity levels.

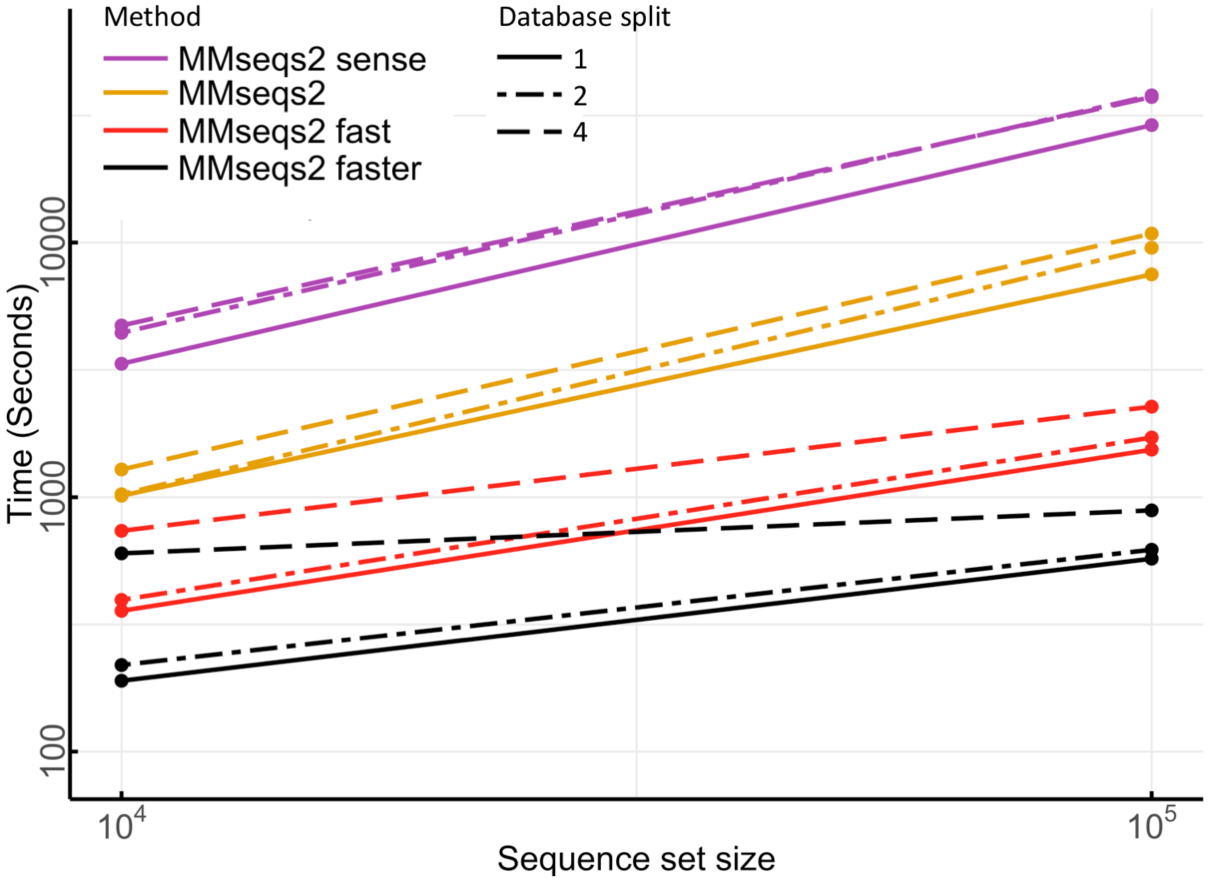

We measured the search time with query sets containing 10000 and 100000 sequences through the UniProt database (Release 2017_03 with 80204488 sequences) using four sensitivity settings (faster, fast, default, and sensitive) and splitting the database into 1, 2, and 4 chunks. The peak memory consumption of the index table for the split levels of 1, 2, and 4 was 190GB, 101GB, and 57GB respectively. All searches ran on a 2×14-core server with 768GB main memory.

Runtime

The prefiltering module is the most time-consuming step. It can scale from minutes in runtime to days by adjusting the sensitivity setting. Searching with 637000 protein sequences against 30 Mio Uniprot sequences took around 12 minutes on 16 cores.

Disk space

The prefiltering results for very large databases can grow to considerable sizes (in the order of TB) of the disk space if very long result lists are allowed and no strict ungapped score threshold is set. As an example, an all-against-all prefiltering run on the 25 Mio sequences with `--max-seqs` 300

yielded prefiltering list with an average length of 150 and an output file size of 78 GB. One entry needs roughly 21 byte of space. To compute the worst case hard disk space usage `S` use the following formula. `N` is the Database sequence size L is `--max-seqs`.

```
S = (21 * N * L) byte
```

Important options for tuning the memory, runtime and disk space usage

- The option `-s` controls the sensitivity in the MMseqs2 prefiltering module. The lower the sensitivity, the faster the prefiltering becomes, though at the cost of search sensitivity. See Set sensitivity -s parameter.
- The option `--max-seqs` controls the maximum number of prefiltering results per query sequence. For very large databases (tens of millions of sequences), it is a good advice to keep this number at reasonable values (i.e. the default value 300). For considerably larger values of `--max-seqs`, the size of the output can be in the range of several TB of disk space for databases containing tens of millions of sequences. Changing `--max-seqs` option has no effect on the run time of the prefilter. Decreasing can speed but alignment but might degrade the sensitivity since the order of the prefilter can differ from the alignment.

Alignment module

In the alignment module, generally only the total runtime and disk space are the critical issues.

Memory consumption

The major part of the memory is required for the three dynamic programming matrices, once per core. Since most sequences are quite short, the memory requirements of the alignment module for a typical database are in the order of a few GB.

Runtime

The alignment is based on a striped vectorized algorithm which can process roughly 2 giga cell updates per second (GCUPS). The time to compute the alignment of two average sized proteins (350 residues) takes roughly 2.0625E-4 seconds on one CPU. For example, computing 23 Mio. alignments on 8 cores takes 2 minutes.

If a huge number of alignments has to be calculated, the run time of the alignment module can become a bottleneck. The run time of the alignment module depends essentially on two parameters:

- The option `--max-seqs` controls the maximum number of sequences aligned with a query sequence. By setting this parameter to a lower value, you accelerate the program, but you may also lose some meaningful results. Since the prefiltering results are always ordered by their significance, the most significant prefiltering results are always aligned first in the alignment module.
- The option `--max-accept` controls the maximum number of alignment results per query sequence.
- The option `--max-rejected` defines the maximum number of rejected sequences for a query until the calculation of alignments stops. The results of the prefilter are aligned from highest ungapped alignment score to lowest.
  We increase a rejection counter after each alignment that does not fulfill the alignment criteria such coverage threshold, e-value threshold etc. If the counter is greater than `--max-rejected` then it stops the alignment and does not take a look at any further prefilter hits. The counter is reset whenever an alignment is accepted. Per default, `--max-rejected` is set to `INT_MAX`, i.e. all alignments until `--max-seqs` alignments are calculated.
- The option `--alignment-mode` controls which part of the alignment should be computed. Alignment mode 1: fastest option is to only compute the score and alignment end position. Alignment mode 2: is slightly slower. It computes alignment start, end, and score. It is roughly 2 times slower than mode 1. Alignment mode 3: alignment start, end, seq.id score. It is roughly 2.5 times slower than mode 1. The option `-a` is as fast as alignment mode 3.

Disk space

Since the alignment module takes the results of the prefiltering module as input, the size of the prefiltering module output is the point of reference. If all hits from the prefilter are accepted by the alignments than the disk space consumption is 1.75 times higher. It needs roughly ~60 byte per record. The option `-a` adds the backtrace and there for increases the size to ~80 byte.

Clustering module

In the clustering module, only the memory consumption is a critical issue.

Memory consumption

The clustering module can need large amounts of memory. The memory consumption for a database containing `N` sequences and an average of `r` alignment results per sequence can be estimated as

```
M = (6 * N * r) byte
```

To prevent excessive memory usage for the clustering of large databases, MMseqs2 uses a cascaded clustering by default, which accumulates sequences per cluster incrementally.

If you run the clustering module separately, you can tune the following parameters:

- `--max-seqs` parameter which controls the maximum number of alignment results per query considered (i.e. the number of edges per node in the graph). Lower value causes lower memory usage and faster run times.
- Alternatively, `-s` parameter can be set to a higher value in order to cluster the database down to higher sequence identities. Only the alignment results above the sequence identity threshold are imported and it results in lower memory usage.

Runtime

Clustering is the fastest step. It needs less than an hour for the clustering of the whole UniProtKB. The core algorithm of cluster mode `--cluster-mode` 0, 1, 2 runs single threaded 3 runs multi threaded.

Disk space

Since only one record is written per cluster, the memory usage is a small fraction of the memory usage in the prefiltering and alignment modules.

Workflows

The resource requirements depend on the combination of modules. Search uses the prefilter and alignment module. Clustering uses the prefilter, alignment and clustering module.
The cascaded clustering sets all the options controlling the size of the output, speed and memory consumption, internally adjusting parameters in each cascaded clustering step.

Each workflow uses a different set of default parameters that can be changed.

How to run MMseqs2 on multiple servers using MPI

MMseqs2 can run on multiple cores and servers using OpenMP and message passing interface (MPI). MPI assigns database splits to each server and each server computes them using multiple cores (OpenMP). Currently `prefilter`, `align`, `result2profile`, `swapresults` can take advantage of MPI. To parallelize the time-consuming k-mer matching and gapless alignment stages prefilter among multiple servers, two different modes are available. In the first, MMseqs2 can split the target sequence set into approximately equal-sized chunks, and each server searches all queries against its chunk.

Alternatively, the query sequence set is split into equal-sized chunks and each server searches its query chunk against the entire target set. The number of chunks is controlled through the `--split` parameter. Splitting the target database is less time-efficient due to the slow, IO-limited merging of results, but it reduces the memory required on each server to:

```
((7 * N * L) / #chunks + 21^k * 8) byte
```

Thus, it allows users to search through huge databases on servers with moderate memory sizes. If the number of chunks is larger than the number of servers, chunks will be distributed among servers and processed sequentially. By default, MMseqs2 automatically decides which mode to pick based on the available memory (assume that all machines have the same amount of memory).

Warning: Make sure that MMseqs2 was compiled with MPI support by using the `-DHAVE_MPI=1` flag during the `cmake` invocation. The precompiled versions of MMseqs2 cannot use MPI (including Conda, Brew, Apt, etc.):

```
mkdir build-mpi && cd build-mpi
cmake -DHAVE_MPI=1 -DCMAKE_BUILD_TYPE=Release ..
```

If MMseqs2 was compiled correctly with MPI support you should see a `-MPI` suffix when you call `mmseqs version`.

To search with multiple server just call the search and add the RUNNER variable. The TMP folder has to be shared between all nodes (e.g. NFS)

```
RUNNER="mpirun -pernode -np 42" mmseqs search queryDB targetDB resultDB tmp
```

For clustering just call the clustering. The TMP folder has to be shared between all nodes (e.g. NFS)

```
RUNNER="mpirun -pernode -np 42" mmseqs cluster DB clu tmp
```

Write temporary files to local disk when running with MPI

As part of its computation, MMseqs2 writes temporary files corresponding to each of the database splits. The number of the database splits is determined by the number of servers and number of cores in each server. By default, temporary files are written to the shared disk.

In case the number of database splits is large, it would mean a high burden of I/O operations on the same disk. To avoid slowdowns due to this issue, an additional parameter can be passed to `search` in MPI mode:

```
RUNNER="mpirun -np 42" mmseqs search queryDB targetDB resultDB tmp --local-tmp
 ↪  /local/hdd/
```

Passing this parameter will write the temporary files of each server (created by its cores) on the indicated path (local disk) and reduce the number of temporary files handled on the shared disk.

How to run MMseqs2 on multiple servers using batch systems

MMseqs2 comes with tools to split database to distribute jobs in batch systems (like sge, slurm, moab, lsf, …). However, the MPI solution is preferred if available since it optimizes the distribution of computing load. Splitting the query database can be used to distribute the load, MMseqs2 has a module called `splitdb` that splits the database in `--split N` chunks. Each can be submitted separate to the grid system.

```
# script to splits the database in 3 parts and submit them to the grid
# split query db
SPLITS=3
QUERYFASTA=queryDB.fasta
QUERYDB=queryDB
mmseqs createdb "${QUERYFASTA}" "${QUERYDB}"
mmseqs splitdb "${QUERYDB}" "${QUERYDB}_split" --split $SPLITS

# create header database to support full mmseqs functionality
# this step can be used if queryDB is used in downstream steps
for file in $(ls "${QUERYDB}_split"_*_$SPLITS); do
    mmseqs createsubdb "${file}.index" "${QUERYDB}_h" "${file}_h"
done

# submit job
split=0
for file in $(ls "${QUERYDB}_split"_*_$SPLITS); do
    bsub mmseqs search "${file}" targetDB aln_${split} tmp
    ((split++))
done
```

Frequently Asked Questions

This section describes common questions.

How to set the right alignment coverage to cluster

MMseqs2 has three modes to control the sequence length overlap "coverage": `--cov-mode` (0) bidirectional, (1) target coverage, (2) query coverage and (3) target-in-query length coverage. In the

context of `cluster` or `linclust`, the query is seen representative sequence and target is a member sequence. The `--cov-mode` flag also automatically sets the `--cluster-mode`.

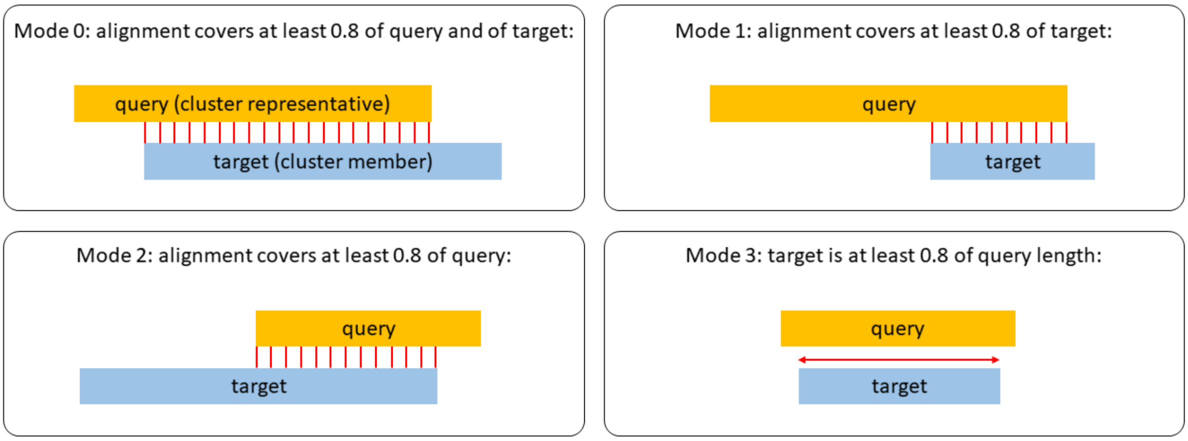

Figure 0.13: Coverage modes in MMseqs2

Bidirectional coverage

With `--cov-mode 0 -c [0.0,1.0]` only sequences are clustered that have a sequence length overlap greater than X% of the longer of the two sequences. This coverage mode should be used to cluster full length protein sequences. The multi domain structure of proteins will be most likely preserved when using a coverage > 80% (`-c 0.8`). Default `--cluster-mode` is the greedy set cover.

For example:

```
q: MAVGTACRPA
t: -AVGTAC---
```

The coverage of this alignment would be 6/10=60%.

```
q: -AVGTAC---
t: MAVGTACRPA
```

The coverage of this alignment would be 6/10=60%.

Target coverage

With `--cov-mode 1 -c [0.0,1.0]` (target-cov mode) only sequences are clustered that have a sequence length overlap greater than X% of the target sequence. The target cov mode can be used to cluster protein fragments. To suppress fragments from becoming representative sequences, it is recommended to use `--cluster-mode 2` in conjunction with `--cov-mode 1`. Default `--cluster-mode` is the greedy incremental clustering (by length).

For example:

```
q: MAVGTACRPA
t: -AVGTAC---
```

The target coverage would be 6/6=100%.

```
q: -AVGTAC---
t: MAVGTACRPA
```

The target coverage would be 6/10=60%.

Query coverage

With `--cov-mode 2 -c [0.0,1.0]` (query-cov mode) only sequences are clustered that have a sequence length overlap greater than X% of the query sequence. The query coverage mode can be used while searching e.g. to assure a certain level of coverage.

For example:

```
q: MAVGTACRPA
t: -AVGTAC---
```

The query coverage would be 6/10=60%.

```
q: -AVGTAC---
t: MAVGTACRPA
```

The query coverage would be 6/6=100%.

How do parameters of CD-HIT relate to MMseqs2

CD-HIT applies a greedy incremental clustering strategy (in MMseqs2 `--cluster-mode 2`) to cluster its sequences. MMSeqs2 automatically picks the optimal clustering strategy based on the coverage mode (`--cov-mode 0` = set cover, `--cov-mode 1,2` = greedy incremental). CD-HIT in default computes the sequence identity globally (`-G 1`), while MMseqs2 computes it locally. But it is possible to mimic the results by applying alignment coverage `--cov-mode X -c 0.X` in CD-HIT `-aS, -aL, -A`.

```
-c 0.XX    -> --min-seq-id 0.XX
-T X       -> --threads X
-M         -> --split-memory-limit
-G 0       -> MMseqs2 uses local alignments is the default, there is no way to make
↪  alignments global
-n         -> -k (MMseqs2 optimizes the k-mer length automatically)
```

```
-s 0.X      -> --cov-mode 4 -c 0.X
-aL 0.X     -> --cov-mode 2 -c 0.X
-aS 0.X     -> --cov-mode 1 -c 0.X
-A 0.X      -> --cov-mode 0 -c 0.X
-g 1        -> it is not possible with MMseqs2 using greedy incremental clustering
 ↪  (needed for --cov-mode 1/2). However, setcover assigns sequence to the best
 ↪  matching representative in default.
-r 1        -> MMseqs2 in default considers forward and reverse strand
```

How does MMseqs2 compute the sequence identity

MMseqs2 computes the sequence identity in two different ways:

(1) When using `--alignment-mode 3` MMseqs2 will compute the number of identical aligned residues divided by the number of aligned columns including columns containing a gap in either sequence.

(2) By default, the sequence identity is estimated from the score per column, i.e., the local alignment bit score divided by the maximum length of the two aligned sequence segments. The estimate uses the linear regression function (shown in red below) between the sequence identity computed as in (1) and the score per column in the scatter plot:

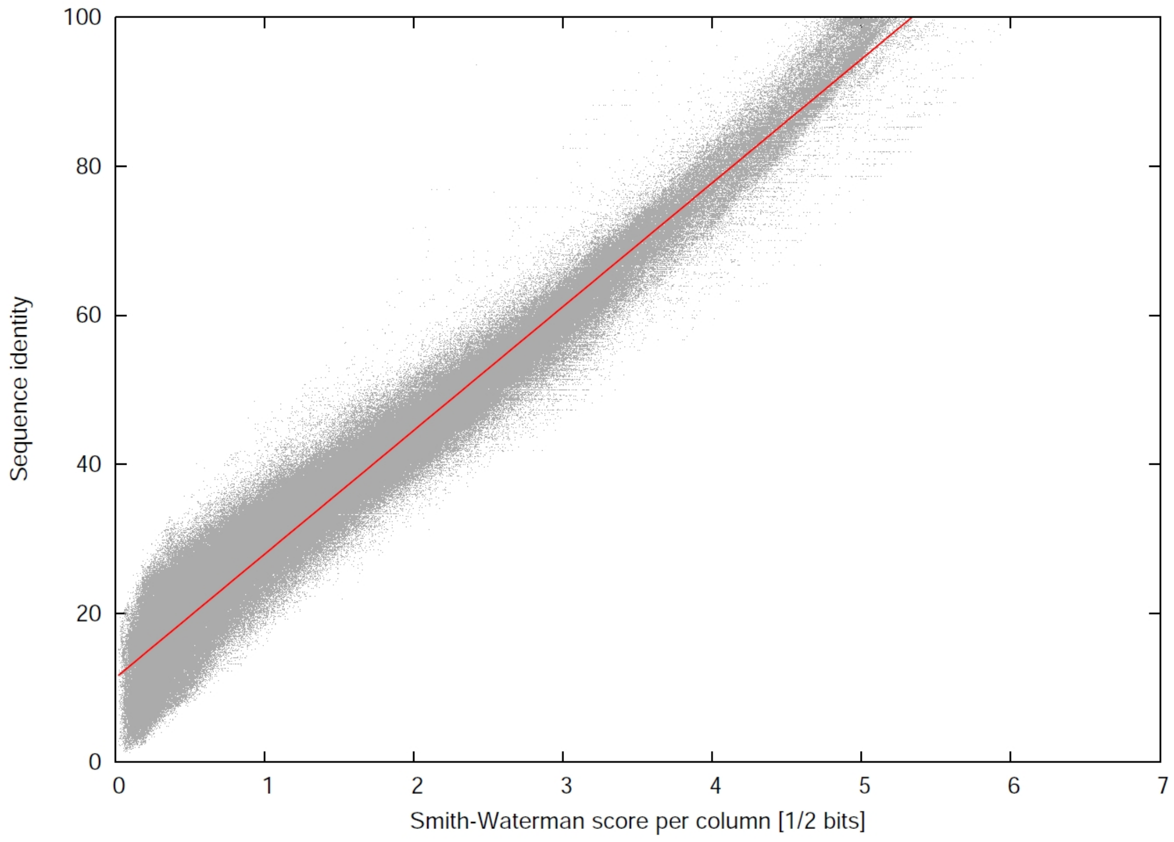

Figure 0.14: Relationship between score per column and sequence identity

The score per column is a better measure of the degree of similarity than the actual sequence identity, because it also takes the degree of similarity between aligned amino acids and the number and length of gaps into account.

How to restart a search or clustering workflow

MMseqs2 checks if files are already computed in the `tmpDir` and skips already computed results. To restart delete temporary result files from the crashing step that were created by MMseqs2 and restart the workflow with the same program call again. You can recognize the temporary files that should be deleted by their file ending `.[0-9]+`.

If the job crashed while merging files they can be merged manually using ffindex_build (https://github.com/soedinglab/ffindex_soedinglab). For example, if the merge step of the alignment fails while using 56 threads then the result could be recovered by using the following command.

```
for i in $(seq 0 55); do ffindex_build -a aln{,.tmp.index} -d aln.$i  -i aln.index.$i
 ↪  ; done
LC_ALL=C sort --parallel 28 -n -k 1,1 aln.tmp.index > aln.index
```

If you change the parameters of the command call than the whole result is recomputed. We have a flag called `--force-reuse` to use the last temporary results also if parameters are changed.

How to control the speed of the search

There are several parameters to improve the search speed. Here is a list of the most important ones:

- `-s` controls how many similar k-mers should be produced during the seeding stage. This is the most important parameter for speed, a lower value is fast but less sensitive and a higher one is sensitive but slower. The default search is already sensitive (default: 5.7)
- `--exact-kmer-matching` turns off similar k-mer generation and performs only an exact k-mer search (default: false)
- `--max-accept` controls how many hits should be accepted by the alignment out of `--max-seqs` (default: 300) prefilter hits. Setting a limit will increase the speed of the alignment (default: 2^15)
- `--max-rejected` aligns until N consecutive alignments are rejected and stops. The rejected counter is reset after a hit was accepted (default: 2^15)
- `--diag-score` this decides if ungapped alignment scoring is enabled. For highly redundant database it can make sense to turn off the ungapped alignment to speed up the prefiltering. This however would also allow more sequences to pass the prefiltering stage (default: true)
- `--min-ungapped-score` the minimum score a prefilter hit in the ungapped alignment stage requires to pass to the alignment stage (default: 15)

How to find the best hit the fastest way

MMseqs2 can apply an iterative approach to speed up best-hit-searches. It will start searching with the lowest sensitivity defined with `--start-sens` and search until the target sensitivity `-s` is reached. The number of steps to reach `-s` can be defined with `--sens-steps`.

Queries are only used again in the next iteration, if no match could be found that fulfilled the acceptance criteria in the previous iteration.

For example, the following search performs three search steps with sensitivity `-s` 1, 4 and 7.

```
mmseqs search qDB tDB rDB tmp --start-sens 1 --sens-steps 3 -s 7
```

Using this iterative approach can speed up best-hit-searches 4-10 times.

There is a chance that the best hit is not found but the chances are low. Prefilter hits found at a lower sensitivity threshold, have more highly conserved k-mers in common. This effect can be reduced if a higher start sensitivity is used (–start-sens 4).

If any hit is good enough add the `--max-accept 1` option to gain a further speedup.

How does MMseqs2 handle low complexity

MMseqs2 uses tantan to reduces low complexity effects on the query and target database.

Query sequences are handled by an amino acid local compositional bias correction. In prefilter and alignment stages we apply a correction to substitution matrix scores assigning lower scores to the matches of amino acids that are overrepresented in the local sequence neighborhood. To switch the compositional bias correction on and off use `--comp-bias-corr`.

Target sequences low-complexity regions are masked during the prefilter stage. We use TANTAN with a threshold of 90% probability for low complexity. Masking can be controlled with `--mask`. Within iterative-profile searches, profiles are also masked at every search iteration. This behavior can be controlled with `--mask-profile`.

How to redundancy filter sequences with identical length and 100% length overlap

To redundancy filter sequences of identical length and 100% overlap `mmseqs clusthash` can be used. It reduces each sequence to a five-letter alphabet, computes a 64 bit CRC32 hash value for the full-length sequences, and places sequences with identical hash code that satisfy the sequence identity threshold into the same cluster.

Example: cluster sequences at 90% sequence identity

```
mmseqs clusthash sequenceDB resultDB --min-seq-id 0.9
mmseqs clust sequenceDB resultDB clusterDB
```

How to add sequence identities and other alignment information to a clustering result

We can add sequence identities and other alignment information to the clustering result `outDB` by running an additional align step:

```
mmseqs cluster sequenceDB resultDb tmp
mmseqs align sequenceDB sequenceDB resultDb alignDB -a
mmseqs convertalis sequenceDB sequenceDB alignDB align.m8
```

The `-a` parameter computes the whole backtrace. `--alignment-mode 3` could be used instead if the backtrace is not needed. This would save disk space. The backtrace is however computed anyway (for the calculation of the sequence identities) and then discarded.

How to run external tools for each database entry

The `apply` module can be used to call an external tool on each entry of a MMseqs2 database. It works like the map step from the map/reduce pattern. It calls for every index entry the specified process with the passed parameters. The process reads the entry data from `stdin` and its `stdout` is written to a new entry in the result database (with the same key). The tool supports OpenMP and MPI parallelization for spreading out the job over several compute nodes.

Example: An awk script which takes an alignment result entry from stdin and prints out all lines with an e-value <0.001 to stdout (Hint: the `filterdb` module can also solve this problem, but with less overhead):

```
mmseqs apply resultDB filteredResultDB -- awk '$4 < 0.001 { print; }'
```

The `apply` module exports the `MMSEQS_ENTRY_NAME` environment variable into the called processes. It contains the current database key.

How to compute a multiple alignment for each cluster

There are two ways to produce MSAs from a clustering

(1) MMseqs2 `mmseqs result2msa` can produce an MSA using a centre star alignment without insertions in the query.

```
mmseqs cluster DB DB_clu tmp
mmseqs result2msa DB DB DB_clu DB_clu_msa
```

(2) The `mmseqs apply` module can be used to call an external multiple aligner. The multiple aligner needs the capability to read stdin and write the result to stdout.

```
mmseqs cluster DB DB_clu tmp
mmseqs createseqfiledb DB DB_clu DB_clu_seq
mmseqs apply DB_clu_seq DB_clu_seq_msa -- clustalo -i - --threads=1
```

How to manually cascade cluster

It is possible to cluster the representative sequences of a clustering run and merge the cluDB results with the following workflow.

```
# first clustering run
mmseqs linclust sequenceDB clu1 tmp1
# create a subset of the sequenceDB only with representative sequences
mmseqs createsubdb clu1 sequenceDB cluSequenceDB
# cluster representative sequences
mmseqs cluster cluSequenceDB clu2 tmp2
# merge two clusterings into one result database
mmseqs mergeclusters sequenceDB final_clu clu1 clu2
```

How to cluster using profiles

The following workflow describes a profile consensus clustering procedure.

(0) Optional: Download a database of reference sequences (or use an existing one instead).

```
mmseqs databases UniRef50 refDB tmp
```

(1) Enrich your sequences against a database of reference sequences:

```
# enrich your database seqDB by searching against a reference database refDB
mmseqs search seqDB refDB resultDB1 tmp -a --num-iterations 2
# turn seqDB into profiles
mmseqs result2profile seqDB refDB resultDB1 profileDB1
```

(2) Cluster profiles by searching the profiles against its consensus sequences

```
# extract consensus sequences from profiles
mmseqs profile2consensus profileDB1 profileDB1_consensus
# search with profiles against consensus sequences of seqDB1
mmseqs search profileDB1 profileDB1_consensus resultDB2 tmp --add-self-matches -a #
↪ Add your cluster criteria here
# cluster the results
mmseqs clust profileDB1 resultDB2 profileDB1_clu
```

How to create a HHblits database

One can turn the output of a search (or clustering) into a HHblits database. You need to have HH-suite properly installed with MPI support. The following procedure creates an HHblits-compatible database “searchMsa” resulting from the enrichment of sequences of “DBquery” with the sequences of “DBtarget”:

```
mmseqs search DBquery DBtarget searchOut tmp -a
mmseqs result2msa DBquery DBtarget searchOut searchMsa --msa-format-mode 1
```

```
# MPI version
mpirun -np 2 cstranslate_mpi -i searchMsa -o searchMsa_cs219 -x 0.3 -c 4 -I ca3m -b
# no MPI version
cstranslate -i searchMsa -o searchMsa_cs219 -f -x 0.3 -c 4 -I ca3m -b
```

The files `/path/to/cs219.lib` and `/path/to/context_data.lib` are provided in the "data" subfolder of your HH-suite installation. The parameters `-x 0.3 -c 4` have been empirically found to perform well.

For creating an HHblits database from a clustering, the procedure is almost the same, except that you have to create symlinks to the ffindex _header and _sequence files needed by HHblits:

```
mmseqs cluster DB clu tmp
mmseqs result2msa DB DB clu cluMsa --msa-format-mode 1
ln -s DB_h cluMsa_header.ffdata
ln -s DB_h.index cluMsa_header.ffindex
ln -s DB cluMsa_sequence.ffdata
ln -s DB.index cluMsa_sequence.ffindex
mpirun -np 2 cstranslate_mpi -i cluMsa -o cluMsa_cs219 -A /path/to/cs219.lib -D
↪ /path/to/context_data.lib -x 0.3 -c 4 -I ca3m -b
```

In the "search" case, those files are generated by MMseqs2, since it needs to merge the query and the target sequence databases. No merging is done for clustering, since both the query and target sequence database are the same.

How to create a target profile database (from PFAM)

Download the latest version of the PFAM in stockholm format:

```
wget http://ftp.ebi.ac.uk/pub/databases/Pfam/current_release/Pfam-A.full.gz
```

Convert stockholm MSAs to a FASTA formatted MSA database.

```
mmseqs convertmsa Pfam-A.full.gz pfam_msa_db
```

Create a profile database in MMseqs2 format.

To turn an MSA into a sequence profile, mmseqs2 needs to decide for each column whether it will be represented by a column in the sequence profile or not. The sensitivity of searches with the sequence profiles can depend critically on which columns are represented. By default, mmseqs2 uses the setting

`--match-mode 0` (profile column assignment by first sequence in MSA),

which means match states are assigned by the first (master) sequence in the MSA: All columns where this master sequence has a residue will be turned into profile columns, all others will be ignored and the residues in them will be modeled as insertions relative to the sequence profile.

This is risky for large Pfam MSAs in which the first sequence might be not very representative of the entire family. A better choice for Pfam is therefore

`--match-mode 1` (profile column assignment by gap fraction)

which turns all columns with at least 50% residues (non-gaps) to profile columns and treats all others as insertions. The threshold ratio can be changed with the option

`--match-ratio 0.5` (change gap fraction threshold for profile column assignment) .

We compute sequence profiles from the FASTA MSAs using

```
mmseqs msa2profile pfam_msa_db pfam_profile --match-mode 1
```

Precompute mmseqs index table (not required for a single search run). Use the `--no-preload` flag later in the search, if the query database is small to medium sized. Without that the precomputed index table will be first read completely into memory (unnecessary overhead).

```
mmseqs createindex pfam_profile tmp -k 5 -s 7
```

Search now against the created profile database:

```
mmseqs search query_db pfam_profile result tmp -k 5 -s 7
```

If your machine has a lot of main memory, use 6-mers for the search (and index building) by specifying `-k 6` instead of `-k 5`. 6-mers provide slightly better sensitivity, however need about 5x more memory (about 25GB instead of 5GB for PFAM, not including the per-thread memory).

How to cluster a graph given as tsv or m8 file

MMseqs2 needs two things to cluster an external graph (1) a sequence database and an (2) result file.

As a first step create your sequence database by calling `createdb` on your input fasta file.

```
mmseqs createdb sequence.fasta sequence
```

It is possible to transform an external TSV in m8 format (BLAST tab) into a result file database using `tsv2db`. The m8 or tsv file must contains a self hit “ID1 ID1 ....” for each entry in the sequence.fasta. Also we need to overwrite the identifier (first and second column) with numerical identifier for the sequence database before calling `tsv2db`. Additonally we provide the parameter `--output-dbtype 5` to indicate that the resulting database is a alignment result database.

```
awk 'NR == FNR {  f[$2] = $1;  next} { line = f[$1]"\t"f[$2]; for(i = 3; i <= NF;
 ↪  i++){ line=line"\t"$i } print line }' sequence.lookup result.m8 > result.m8.newid
mmseqs tsv2db result.m8.newid result --output-dbtype 5
```

Now we should be able to use the internal clustering (greedy incremental, connected component, set cover) algorithm of MMseqs2

```
mmseqs clust sequence result clu
```

How to search small query sets fast

MMseqs2 can perform single queries fast if the whole index fits into memory by using memory-mapped file I/O (mmap). If the target database is not in memory, MMseqs2 needs to load GBs from the hard disk for each query, which will take minutes. The following workflow creates an index and fetches the database into memory and efficiently searches through it.

First we need to setup the database by creating a database with `createdb`, index it `createindex` and then load the index into memory `touchdb` or `vmtouch` (https://github.com/hoytech/vmtouch).

```
mmseqs createdb targetDB.fasta targetDB
mmseqs createindex targetDB tmp
mmseqs touchdb targetDB
# alternative using vmtouch
vmtouch -l -d -t targetDB.idx
```

Once the database is in memory it is possible to run instant searches against it by using the `--db-load-mode 2`

```
mmseqs search queryDB targetDB aln tmp --db-load-mode 2
mmseqs convertalis queryDB targetDB aln aln.m8 --db-load-mode 2
```

The `touchdb` module fetches the precomputed index database into memory and `--db-load-mode 2` tells MMseqs2 to `mmap` the database instead of copying the whole precomputed index into memory. This saves, for a large database, minutes of copying from the storage system into RAM. However, this is less efficient for large query sets.

The reason for this is that copying the index into memory will use transparent huge pages (on operating systems that support THP), which in turn results in less TLB cache misses during the prefiltering stage. For a large query set the use of THP results in 15-20% speed improvement of the prefiltering stage.

What is the difference between the map and search workflow

The `map` and `search` workflow both call the `search` workflow. The only difference are the default parameters and alignment strategy. `map` should be used for high identities which is more of a technical task rather than measuring homology. Therefore, we turned off compositional bias correction, we do not mask the database, search with low sensitivity `-s 2` and we align only ungapped.

How to build your own MMseqs2 compatible substitution matrices

You can use the (still Work in Progress) R script in `util/format_substitution_matrix.R` to prepare a substitution matrix for use in MMseqs2. It takes a matrix from `stdin` and returns the reformatted matrix to `stdout`.

```
util/format_substitution_matrix.R < data/MATRIX.mat > data/MATRIX.out
mmseqs search query db res tmp --sub-mat data/MATRIX.out
```

How to create a fake prefiltering for all-vs-all alignments

The following shell function can be used to create a fake prefiltering result for all-vs-all-alignments. This works since the target database index contains a list of all targets and the alignment module requires a tab separated list of lines beginning with target keys. A newly created index will then repeatedly point to the same list of target keys. However, this function will not work with nucleotide-nucleotide searches, since we need to have a valid diagonal for the banded alignment.

```
fake_pref() {
    QDB="$1"
    TDB="$2"
    RES="$3"

    # create link to data file which contains a list of all targets that should be
     ↪  aligned
    ln -s "${TDB}.index" "${RES}"
    # create new index repeatedly pointing to same entry
    INDEX_SIZE="$(echo $(wc -c < "${TDB}.index"))"
    awk -v size=$INDEX_SIZE '{ print $1"\t0\t"size; }' "${QDB}.index" > "${RES}.index"
    # create dbtype (7)
    awk 'BEGIN { printf("%c%c%c%c",7,0,0,0); exit; }' > "${RES}.dbtype"
}
```

Example usage:

```
fake_pref qdb tdb allvsallpref
mmseqs align qdb tdb allvsallpref allvsallaln
mmseqs convertalis qdb tdb allvsallaln allvsall.m8
```

How to compute the lowest common ancestor (LCA) of a given set of sequences

If you already have sets of sequences that you want to compute LCAs of you can do that by following these steps. First we prepare all input (with examples given): 1. FASTA file containing all sequences (`sequences.fasta`):

```
>sp|A7ZUJ8|RL10_ECO24 50S ribosomal protein L10 OS=Escherichia coli O139:H28 (strain
 ↪ E24377A / ETEC) OX=331111 GN=rplJ PE=3 SV=1
MALNLQDKQAIVAEVSEVAKGALSAVVADSRGVTVDKMTELRKAGREAGVYMRVVRNTLL
RRAVEGTPFECLKDAFVGPTLIAYSMEHPGAAARLFKEFAKANAKFEVKAAAFEGELIPA
SQIDRLATLPTYEEAIARLMATMKEASAGKLVRTLAAVRDAKEAA
>sp|P0A7J6|RL10_SHIFL 50S ribosomal protein L10 OS=Shigella flexneri OX=623 GN=rplJ
 ↪ PE=3 SV=2
MALNLQDKQAIVAEVSEVAKGALSAVVADSRGVTVDKMTELRKAGREAGVYMRVVRNTLL
RRAVEGTPFECLKDAFVGPTLIAYSMEHPGAAARLFKEFAKANAKFEVKAAAFEGELIPA
SQIDRLATLPTYEEAIARLMATMKEASAGKLVRTLAAVRDAKEAA
>sp|Q1C1T9|RL10_YERPA 50S ribosomal protein L10 OS=Yersinia pestis bv. Antiqua (strain
 ↪ Antiqua) OX=360102 GN=rplJ PE=3 SV=1
MALNLQGKQAIVAEVKEVAKGALSAVVADSRGVTVDKMTELRRAGREAGVHMQVVRNTLL
RRIVEGTPFECLKDTFVGPTLIAFSAEHPGAAARLFKAFAKDNAKFEVKAAAFEGELIPA
AQIDRLATLPTYEEAIARLMGTMKEAAAGKLVRTLAALRDQKEAA
```

2. TSV file with accessions (see Identifier parsing) in the first column and numeric NCBI taxon identifiers in the second column (`taxonomy.tsv`):

```
A7ZUJ8  331111
P0A7J6  623
Q1C1T9  360102
```

3. TSV file with adjecency list of sequences forming the sets (`sets.tsv`):

```
A7ZUJ8  A7ZUJ8
A7ZUJ8  P0A7J6
A7ZUJ8  Q1C1T9
```

Next, we create a sequence database with taxonomy information:

```
mmseqs createdb sequences.fasta seqdb
mmseqs createtaxdb seqdb tmp --tax-mapping-file taxonomy.tsv
```

This will also download the latest taxonomy dump from the NCBI. You can specify your own with the `--ncbi-tax-dump` parameter.

We replace the accessions with the internal MMseqs2 numeric entry keys:

```
awk 'NR == FNR { f[$2] = $1; next; } { line = f[$1]"\t"f[$2]; for(i = 3; i <= NF; i++)
↪  { line=line"\t"$i } print line; }' seqdb.lookup sets.tsv > sets_numerickeys.tsv
```

And turn this database into a kind of clustering result that can be read by other MMseqs2 modules:

```
mmseqs tsv2db sets_numerickeys.tsv setsdb --output-dbtype 6
```

With everything set up, we can compute lowest common ancestors of each given set (in this case a single set):

```
mmseqs lca seqdb setsdb lcadb
```

This database can now be converted back to a TSV file:

```
mmseqs createtsv seqdb lcadb lca.tsv
```

The resulting file contains for each set a line with a taxonomic assignment with the set representative accession in the first column (see Taxonomy output and TSV):

```
A7ZUJ8  91347   order   Enterobacterales
```

Workflow control parameters

Search workflow

Compares all sequences in the query database with all sequences in the target database.

Usage:

```
mmseqs search <queryDB> <targetDB> <outDB> <tmpDir> [opts]
```

Options:

```
-s [float] Target sensitivity in the range [1:7.5] (default=5.7).
```

Adjusts the sensitivity of the prefiltering and influences the prefiltering run time. 1.0 fastest - 8.5 sensitive. The sensitivity between 8 to 8.5 should be as sensitive as BLAST. For detailed explanation see section Computation of Prefiltering Scores using `mmseqs prefilter`.

Clustering workflow

Calculates the clustering of the sequences in the input database.

Usage:

`mmseqs cluster <sequenceDB> <outDB> <tmpDir> [opts]`

Options:

`--single-step-clustering Starts the single-step instead of the cascaded clustering workflow.`

The database can be clustered in a single step instead of with a cascaded workflow. This increases runtime and memory requirements substantially and decreases sensitivity, but guarantees, that all cluster members strictly fulfill the selection criteria, such as sequence identity or coverage. After merging of clusters in the cascaded clustering, the e.g. sequence identity of the representative with the members of the to be merged cluster, might fall under the given sequence identity threshold.

`-s [float] Target sensitivity in the range  [1:7.5] (default= depended on the sequence identity).`

Adjusts the sensitivity of the prefiltering and influences the clustering run time. For detailed explanation see section Computation of Prefiltering Scores using `mmseqs prefilter`. If `-s` is not set then we determin it automatically based on `--min-seq-id`.

```
How MMseqs2 sets -s sensitivity based on --min-seq-id
minSeqId <= 0.3 =  6.0
minSeqId <= 0.8 && minSeqId > 0.3  = 1.0 + (1.0 * (0.7 - minSeqId) * 10)
minSeqId > 0.8 =  1.0
```

`--min-seq-id [float] list matches above this sequence identity [0.0:1.0] (default=0.0).` Read more about how MMseqs2 computes sequence identity in section How does MMseqs2 compute the sequence identity.

`--cov-mode [int] "0: coverage of query and target, 1: coverage of target [0:1] (default=0).  -c [float] "list matches above this fraction of covered residues (see cov-mode) [0.0:1.0] (default=0.8).` Read more about how coverage is computed in section How to set the right alignment coverage to cluster.

Updating workflow

Updates the existing clustering of the previous database version with new sequences from the current version of the same database.

Usage:

`mmseqs clusterupdate <oldDB> <newDB> <oldDB_clustering> <outDB> <tmpDir> [opts]`

Options:

`--sub-mat [file] Amino acid substitution matrix file.`

Substitution matrices for different sequence diversities in the required format can be found in the MMseqs2 `data` folder.

Environment variables used by MMseqs2

- `MMSEQS_FORCE_MERGE` Define to disallow MMseqs2 to produce split data files (`data.0`, …, `data.N`) for databases output.
- `MMSEQS_NUM_THREADS` Set maximum number of threads MMseqs2 will use. Overwrites `--threads`.
- `MMSEQS_IGNORE_INDEX` Ignore precomputed index (`.idx`) if present.
- `RUNNER` See How to run MMseqs2 on multiple servers using MPI.
- `TTY` Undefined: MMseqs2 will automatically determine if it's running in an interactive session and enable color output and enhanced progress bars. 0: Force disable color output and enhanced progress bar. 1: Force enable color output and enhanced progress bar.

External libraries used in MMseqs2

We would also like to thank the developers of the open source libraries used in MMseqs2:

- Striped Smith-Waterman Library
- ALP Library
- TANTAN
- Open MP Template Library
- ksw
- kseq
- itoa
- blast2lca
- sse2neon+DLTcollab fork+SIMDe
- zstd
- tinyexpr
- Krona
- xxhash

- microtar
- nedmalloc
- ips4o

License terms

The software is made available under the terms of the GNU General Public License v3. Its contributors assume no responsibility for errors or omissions in the software.
